# A Novel Ruthenium(II) Complex With Lapachol Induces G2/M Phase Arrest Through Aurora-B Kinase Down-Regulation and ROS-Mediated Apoptosis in Human Prostate Adenocarcinoma Cells

**DOI:** 10.3389/fonc.2021.682968

**Published:** 2021-06-24

**Authors:** Rone A. De Grandis, Katia M. Oliveira, Adriana P. M. Guedes, Patrick W. S. dos Santos, Alexandre F. Aissa, Alzir A. Batista, Fernando R. Pavan

**Affiliations:** ^1^ School of Pharmaceutical Sciences, São Paulo State University, Araraquara, Brazil; ^2^ School of Medicine, University of Araraquara, Araraquara, Brazil; ^3^ Department of Chemistry, Federal University of São Carlos, São Carlos, Brazil; ^4^ School of Medicine of Ribeirão Preto, University of São Paulo, Ribeirão Preto, Brazil; ^5^ Department of Biochemistry and Molecular Genetics, University of Illinois at Chicago College of Medicine, Chicago, IL, United States

**Keywords:** lapachol, naphtoquinones, 3D-cell culture, DNA damage (comet assay), ROS - reactive oxygen species, apoptosis

## Abstract

Lapachol is a well-studied natural product that has been receiving great interest due to its anticancer properties that target oxidative stress. In the present work, two novel lapachol-containing ruthenium(II) complexes [Ru(Lap)(dppm)(bipy)]PF_6_ (**1**) and [Ru(Lap)(dppm)(phen)]PF_6_ (**2**) [Lap = lapachol, dppm = 1,1′-bis(diphosphino)methane, bipy = 2,2′-bipyridine, phen = 1,10-phenantroline] were synthesized, fully characterized, and investigated for their cellular and molecular responses on cancer cell lines. We found that both complexes exhibited a potent cytotoxic effect in a panel of cancer cell lines in monolayer cultures, as well as in a 3D model of multicellular spheroids formed from DU-145 human prostate adenocarcinoma cells. Furthermore, the complex (**2**) suppressed the colony formation, induced G2/M-phase arrest, and downregulated Aurora-B. The mechanism studies suggest that complex (**2**) stimulate the overproduction of reactive oxygen species (ROS) and triggers caspase-dependent apoptosis as a result of changes in expression of several genes related to cell proliferation and caspase-3 and -9 activation. Interestingly, we found that N-acetyl-L-cysteine, a ROS scavenger, suppressed the generation of intracellular ROS induced by complex (**2**), and decreased its cytotoxicity, indicating that ROS-mediated DNA damage leads the DU-145 cells into apoptosis. Overall, we highlighted that coordination of lapachol to phosphinic ruthenium(II) compounds considerably improves the antiproliferative activities of resulting complexes granting attractive selectivity to human prostate adenocarcinoma cells. The DNA damage response to ROS seems to be involved in the induction of caspase-mediated cell death that plays an important role in the complexes' cytotoxicity. Upon further investigations, this novel class of lapachol-containing ruthenium(II) complexes might indicate promising chemotherapeutic agents for prostate cancer therapy.

## Introduction

Prostate cancer is the second most frequent malignancy (after lung cancer) in the male population and rising incidences have been observed worldwide (1, 2). The mortality rate rises with age, and almost 55% of all deaths occur after 65 years of age (3).

The current chemotherapeutic drugs have clinically serious toxicities. Furthermore, anti-androgen drugs, designed to disrupt the function of androgens, have been linked to the onset of drug-resistant prostate cancer (4). Therefore, the development of novel chemotherapy agents remains a requirement. In the search for new anticancer drugs, several promising discoveries have been accomplished using natural products, such as naphthoquinone derivatives.

Lapachol (2-hydroxy-3-(3-methylbut-2-en-1-yl)naphthalene-1,4-dione) is a naphthoquinone, which was originally isolated from species of the Bignoniaceae family (5). We, along with other research groups, have been investigating the anticancer potential of lapachol and other naphtoquinones, free or coordinated to a metal (6–10). The presence of quinone group in these molecules is responsible for their outstanding known antineoplastic characteristics, including cytotoxicity, genotoxicity, and potent antitumor properties *in vivo* (11).

Historically, much attention has been given to naphtoquinones after its ability to directly target DNA topoisomerases and inhibit their activity, which results in cytotoxicity (12). Recognized studies also demonstrated that lapachol can generate semiquinone radicals by bioreduction in intracellular hypoxic conditions, which is involved with the production of reactive oxygen species (ROS) selectively in cancer cells (13, 14). Later, the anticancer potential of lapachol and its analogs have been extensively explored (15, 16). These studies reported the ability of lapachol to induce mitochondria-mediated cellular apoptosis by activating caspases and PARP (17). Lapachol analogs also downregulate the expression of the c-Myc, cyclin D1 and cyclin B1, which induce cell cycle arrest in the S and G2/M phases resulting in inhibition of tumor cell proliferation (18).

Recent studies have shown that the coordinating ability of several organic molecules toward transition metals can lead to a successful rational design of metallodrugs. In this field, ruthenium-based complexes are highlighted due to the unique properties of this metal, such as chemical stability, a variety of oxidation states, structural diversity, and low toxicity *in vivo*. Furthermore, ruthenium complexes under pre-clinical and clinical studies are showing promising results, representing a possible novel class of less toxic antineoplastic chemotherapy (19–21).

**Figure 1:**
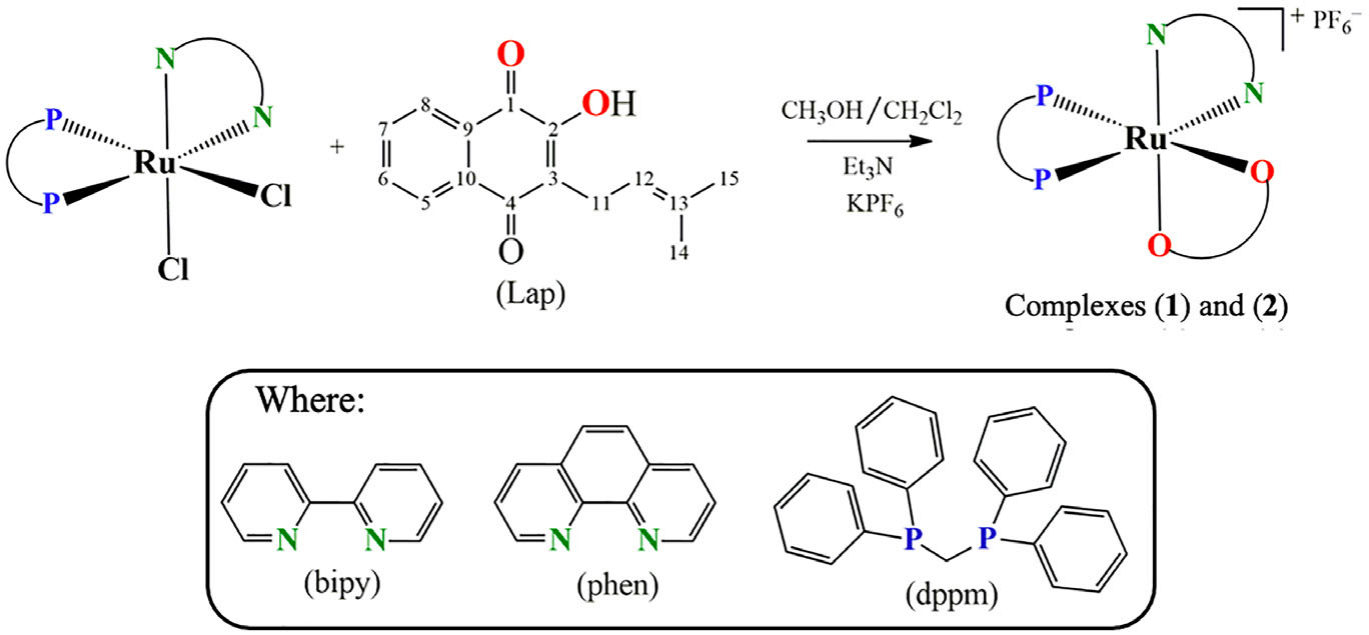
Route for the synthesis of complexes [Ru(Lap)(dppm)(bipy)]PF_6_ **(1)** and [Ru(Lap)(dppm)(phen)]PF_6_ **(2)**.

Hence, within the framework of our continuous effort to design novel metallodrug candidates, in the last years, our research group has synthesized several ruthenium complexes with promising pharmacological activities for the anticancer purpose (22–26). In the light of those findings, herein we have investigated for once the cellular and molecular responses of two novel lapachol-containing ruthenium(II) complexes [Ru(Lap)(dppm)(bipy)]PF_6_ (**1**) and [Ru(Lap)(dppm)(phen)]PF_6_ (**2**). The corresponding complexes (**Figure 1**) were synthesized and characterized, and their potential as anticancer agents was investigated on human prostate adenocarcinoma cells.

## Material and Methods

### Synthesis and Characterization of Novel Lapachol-Containing Ruthenium Complexes

All procedures involving solutions of the complexes were performed under inert argon atmosphere, and all reagents used were of analytical or pure grade, and the solvents were dried using appropriate agents. The RuCl_3_.nH_2_O, *bis*(diphenylphosphine)methane (dppm), 2,2'-bipyridine (bipy), 1,10-phenanthroline (phen), and KPF_6_ were purchased from Sigma-Aldrich MERCK (St. Louis, MO, USA) and used as received. Lapachol (Lap) was extracted from the bark of *Tabebuia aurea* (Manso) S. Moore as reported in the literature (27).

The ^1^H, ^13^C{^1^H} and ^31^P{^1^H} NMR experiments were recorded on a 9.4 T Bruker Avance III 400 MHz spectrometer using a 5-mm internal diameter indirect probe with Automatic Tuning Matching. Electrochemical assays were executed using a Bioanalytical Systems Inc, model BAS-100B/W at room temperature in CH_2_Cl_2_ containing 0.10 M Bu_4_NClO_4_ (TBAP) (Fluka Purum) as a support electrolyte. The Ag/AgCl (0.10 M Bu_4_NOH in CH_2_Cl_2_) in a Luggin capillary probe was used as reference electrode and platinum foils as working and auxiliary electrodes. The IR spectra were acquired using a FT-IR Bomem-Michelson 102 spectrometer using KBr pellets. Elemental analysis (C, H, and N) was determined using an CHNS Element Analyzer (Thermo Fischer Scientific, Fisons EA-1108 model). UV-vis spectra were recorded in a Hewlett Packard diode array—8452A spectrophotometer. Conductivity was acquired using a MeterLab CDM2300 at room temperature.

The precursors complexes with general formula *cis*-[RuCl_2_(dppm)(N-N)], where N-N = 2,2'-bipyridine (bipy) or 1,10-phenanthroline (phen) and dppm = *bis*(diphenylphosphine)methane, were synthesized according to described in the literature (23, 28, 29).

### [Ru(Lap)(dppm)(bipy)]PF_6_ (1)

To a Schlenk flask containing 40 ml of dichlorometane and 40 ml of acetone (1:1, v/v), the *cis*-[RuCl_2_(dppm)(bipy)] (0.14 mmol), lapachol (0.21 mmol), KPF_6_ (0.28 mmol), and 50 µl of triethylamine were added. The reaction was maintained in reflux under argon atmosphere for 12 h. After that, the volume of the mixture was reduced to ca. 3 ml, and the complex precipitated by addition of 1 ml of water. The solid was filtered off, washed with water, diethyl ether and dried under vacuum. Yield: 0.109 g (76%). Elemental analysis for [C_50_H_43_F_6_N_2_O_3_P_3_Ru]: exp. (calc): C, 58.85 (58.43), H, 4.40 (4.22), and N, 2.85 (2.73). Λ_M_ = 59 Ω^−1^ cm^2^ mol^−1^, in 1.0 mM CH_2_Cl_2_ solution. IR (cm^-1^): *v*(C_1_=O) 1546, *v*(C_4_=O) 1608, *v*(C_2_–O) 1103, *v*(P–F) 840 and 557, *v*(Ru–O) 510 and *v*(Ru-N) 483. ^31^P{^1^H} NMR (162 MHz, CH_2_Cl_2_, 298 K): δ (ppm) 18.2; 6.2/14.7; 5.8/*^2^J* = 76.1/66.4, –144 (1P, hept, $PF_{6}^{-}$, *J*_PF_ = 711 Hz). ^1^H NMR (400 MHz, DMSO-d_6_, 298 K): δ (ppm) 8.7–6.6 (m, 32H, an overlap of aromatic protons of dppm (20 H), bipy (8H), and Lap (4H)), 5.4 (m, 1H, Lap), 4.8 (m, 2H, dppm), 3.1–2.8 (m, 2H, Lap), 1.4–1.3 (m, 6H, 2CH_3_ of Lap). ^13^C{^1^H} NMR (125.74 MHz, DMSO-d_6_, 298 K): δ(ppm) 196.5 (C_1_=O), 181.4 (C_4_=O), 169.6 (C_2_–O).

### [Ru(Lap)(dppm)(phen)]PF_6_ (2)

To a Schlenk flask containing 40 ml of dichlorometane and 40 ml of acetone (1:1, v/v), the *cis*-[RuCl_2_(dppm)(phen)] (0.17 mmol), lapachol (0.21 mmol), KPF_6_ (0.28 mmol), and 50 µl of triethylamine were added. For the synthesis of this complex, the same procedure of complex (**1**) was used. Yield: 0.107 g (75%). Elemental analysis for [C_52_H_43_F_6_N_2_O_3_P_3_Ru]: exp. (calc): C, 59.71 (59.37); H, 4.39 (4.12); and N, 2.95 (2.66). Λ_M_ = 38.27 Ω^−1^ cm^2^ mol^−1^, in 1.0 mM CH_2_Cl_2_ solution. IR (cm^−1^): *v*(C_1_=O) 1547, *v*(C_4_=O) 1610, *v*(C_2_–O) 1097, *v*(P-F) 837, and 555, *v*(Ru-O) 508 and *v*(Ru-N) 480. ^31^P{^1^H} NMR (162 MHz, CH_2_Cl_2_, 298 K): δ (ppm) 18.3; 7.1/14.9; 6.5/*^2^J* = 74.5/68.0, −144 (1P, hept, $PF_{6}^{-}$, *J*_PF_ = 711 Hz). ^1^H NMR (400 MHz, DMSO-d_6_, 298 K): δ (ppm) 9.0–6.3 (m, 32H, an overlap of aromatic protons of dppm (20 H), phen (8H), and Lap (4H)), 5.5 (m, 1H, Lap), 4.8 (m, 2H, dppm), 3.2–2.8 (m, 2H, Lap), 1.8–1.7 (m, 6H, 2CH_3_ of Lap). ^13^C{^1^H} NMR (125.74 MHz, DMSO-d_6_, 298 K): δ(ppm) 196.8 (C_1_=O), 181.8 (C_4_=O), 169.6 (C_2_–O).

### Partition Coefficient (*n-*Octanol/Water) Determination

The octanol-water partition coefficients (log *P*) were quantified using the shake-flask method (30). Each complex was tested in a mixture of equal volumes of water and *n*-octanol with continuous shaking for 24 h at 112*g* and 37°C. Then the samples were centrifuged for 5 min at 10*g*, and the organic and aqueous phases were separated. The concentration of complex in each phase was measured spectrophotometrically to determine values of log *P =* [compound] (in *n*-octanol)/[compound] (in water). The experiments were carried out in triplicate.

### Cell Lines and Culture Conditions

Human lung carcinoma (A549, ATCC^®^ CCL-185^™^), human hepatocellular carcinoma (HepG2, ATCC^®^ HB-8065^™^), and human breast adenocarcinoma (MDA-MB-231, ATCC^®^ HTB-26^™^) were obtained from American Type Cell Collection (ATCC). Human melanoma (A-375, BCRJ-0278), Human colorectal adenocarcinoma (Caco-2, BCRJ 0059), human prostate adenocarcinoma (DU-145, BCRJ-0078, and PC-3, BCRJ-0269), human mouth fibroblast (FGH, BCRJ-0089), and human prostate epithelial cells (PNT-2, BCRJ-0366) were obtained from Rio de Janeiro Cells Bank (BCRJ). The cells A549, A-375, Caco-2, DU-145, HepG2, MDA-MB-231, and FGH were cultured in Dulbecco’s Modified Eagle’s Medium (DMEM, Gibco-Invitrogen^®^), PC-3 and PNT-2 cells were grown in Roswell Park Memorial Institute Medium (RPMI-1640, Gibco-Invitrogen^®^). Both mediums were supplemented according to the needs of each lineage, as recommended by its cell banks with 2 mM l-glutamine (Gibco-Invitrogen^®^), 10% or 20% fetal bovine serum (Gibco-Invitrogen^®^), 1% antibiotic-antimycotic solution (5 mg/ml penicillin, 5 mg/ml streptomycin, and 10 mg/ml neomycin, Gibco-Invitrogen^®^), and 3.7 g/L of NaHCO_3_ (Sigma-Aldrich). All cell lines were cultured in flasks at 37°C in 5% CO_2_ and 96% of relative humidity according to the procedures proposed by (31), and a mycoplasma stain kit (Sigma-Aldrich) was used to confirm the use of cells devoid of contamination. The assays were conducted between the third and the seventh-cell passage with sub-cultures every 3 to 4 days to maintain exponential growth. The cell viability was checked using the trypan blue dye exclusion assay for all experiments where over 95% of the cells were viable at the beginning of the tests.

### Cytotoxicity Activity Assay

The *in vitro* antiproliferative activity was quantified using the Alamar blue^®^ assay, according to the method reported by (32). Cells were seeded in 96-well plates for all experiments (1.5 × 10^4^ cells/well). After 24 h, the complexes were dissolved in DMSO and added to each well and incubated for 24 h. Dilutions of the complexes were prepared to obtain concentrations ranging from 0.3 to 100 μM. Cisplatin (Fauldcispla^®^) and doxorubicin hydrochloride (Fauldoxo^®^) were used as the reference cytotoxic drugs. DMSO (0.1% v/v) was used as the vehicle control. Following 24 h of incubation, 50 μl of Alamar blue^®^ (resazurin at 0.01% w/v, Sigma-Aldrich) was added to each well, and the plates were incubated, at 37°C, in the dark. Fluorescence reading was performed in a Synergy H1 Fluorescence Spectrophotometer (BioTek^®^), using excitation and emission filters at wavelengths of 560 and 590 nm, respectively. The fluorescence intensity was measured in arbitrary fluorescence units (AFU) and the AFU of untreated cells (CTL) was considered 100%. The cytotoxic effects of the compounds were estimated in terms of cell proliferation inhibition (%) and expressed as half-maximal inhibitory concentration (IC_50_). The cell proliferation inhibition percentage of cells exposed to treatments was calculated following: % Inhibition = 100 − (AFU of treated well/AFU of control well) × 100). The IC_50_ values were calculated from the cell proliferation inhibition percentage by nonlinear regression method using CompusSyn^®^ Software.

### 3D Multicellular Tumor Spheroids Culture

Multicellular tumor spheroids (MCTSs) were obtained according to the method reported by (33). Briefly, 200 μl of a solution of DU-145 cells (2.0 × 10^3^ cells/well) were inserted in agarose-coated 96-well plates (1.5% w/v) and cultured in complete medium plus 3% Matrigel^®^ (Corning). DU-145-MCTSs with stable structures had formed after five days. Then, spheroids were exposed to the complexes in a range of five different concentrations varying from 0.25 to 4.0 μM and incubated for 24 h. Cisplatin (Fauldcispla^®^) at 25 μM was used as the reference cytotoxic drug. Negative control received the vehicle (DMSO 0.1% v/v) that was used for diluting the compounds tested. Finally, the MCTS proliferation inhibition (%) and IC_50_ were determined by Alamar blue^®^ assay as described above. To investigate morphological changes other MCTSs were treated with 0.25, 0.5, 1.0, 1.5, and 2.0 μM of complex (**2**) and examined for 7 days. Spheroid integrity and diameter of MCTSs were analyzed by phase-contrast microscopy (Inverted Trinocular Microscope Opton TNB-O5T-PL) using AxioVision LE software (Carl Zeiss, Germany).

### Clonogenic Assay

The clonogenic assay was performed in accordance with the guidelines of (34). The DU-145 cells (1 ×10^6^cells/well) were inserted in six-well plate and treated with different concentrations varying of 0.125 to 1.5 μM of both complexes. Cisplatin (Fauldcispla^®^) and doxorubicin hydrochloride (Fauldoxo^®^) were used as the reference cytotoxic drugs. DMSO (0.1% v/v) was used as the vehicle control. After 24 h, the cells were washed with PBS (Gibco-Invitrogen^®^) and harvested by trypsinization. Cells were counted using a Neubauer chamber and replated then on cell culture dishes of 21.5 cm^2^ in a density of 200 cells per culture dish for each treatment in triplicate. After 7 days, cells were fixed in ice-cold methanol: acetic acid: distilled water (1:1:8), air-dried, and stained with crystal violet (0.5% w/v, Sigma-Aldrich) for 20 min. Colonies with at least 50 cells were counted. Plating efficiency (PE) of DU-145 cells was calculated by the ratio of the number of colonies observed after 7 days of incubation to the number of cells seeded (two hundred). The average of colonies in control was considered as 100%, and the number of colonies that arise after treatment of cells is called the surviving fraction (SF), obtained by:

$$SF\;=\;\frac{Numberofcoloniescountedineachtreatment}{Numberofcoloniescountedincontrol\;x\;PE}\;\times \;100$$

### Cell Cycle Analysis

DNA staining was performed using BD Cycletest^™^ Plus DNA Kit (BD Biosciences, USA) according to the manufacturer’s instructions. Briefly, DU-145 cells were seeded (5.0 × 10^4^ cells/well) into 24-well culture plates and maintained to attach at 37°C in 5% CO_2_. After 24 h, the culture medium was replaced by complete culture medium containing 0.25, 0.5, 1.0, and 1.5 μM of complex (**2**). DMSO (0.1% v/v) was used as the vehicle control. Then, after 24 h treatment, the cells were harvested and fixed with 70% ethanol at 4°C for 1 h. Cells were incubated with 0.1 mg/ml RNase and 0.5 mg/ml propidium iodide (PI) for 10 min. Samples were then filtered using 50-µm nylon mesh and the FACSCanto flow cytometer (Becton Dickinson, Franklin Lakes, NJ, USA) was used to analyze the DNA histogram. Data from 10,000 cells were acquired and analyzed using the ModFit software (BD Biosciences, USA). The results were expressed as percentage of cells in each cell cycle phase (G1, S, and G2/M).

### Annexin-V/PI Staining Assay

The measurement of cell death was performed by the guidelines of (35). Detection of apoptotic/necrotic cells was determined by flow cytometry using the Alexa Fluor^®^ 488 Annexin V/PI (Thermo Fischer Scientific), and the analyses were performed according to the manufacturer’s instructions. Briefly, DU-145 cells were seeded (5.0 × 10^4^ cells/well) in 12-well plates and maintained to attach at 37°C in 5% CO_2_ for 24 h. After, cells were treated with 0.25, 0.5, 1.0, and 1.5 μM of both complexes. Cisplatin (Fauldcispla^®^) and doxorubicin hydrochloride (Fauldoxo^®^) were used as the reference cytotoxic drugs. DMSO (0.1% v/v) was used as the vehicle control. Following 24 h of incubation, the cells were collected with Accutase^®^ (Gibco-Invitrogen^®^), washed with PBS and resuspended in 200 μl of cold annexin-binding buffer. Next, 10 μl of Alexa Fluor^®^ was added to Annexin V (50 μl/ml) staining buffer, and the mixture was incubated in the dark at 4°C for 15 min. Previously, the analyses, 100 μl of PI (2 μg/ml) was added, and then, the fluorescence was measured by flow cytometry in a FACSCanto flow cytometer (BD Biosciences, USA) using the Diva software. Ten thousand events were evaluated per experiment, and cellular debris was omitted from the analysis.

### Measurement of Cellular Reactive Oxygen Species Levels

Total intracellular ROS generation was measured using 2′,7′-dichlorodihydrofluorescein diacetate (H_2_DCFDA, Sigma-Aldrich), as reported by (36). Briefly, DU-145 cells were seeded (1.5 × 10^4^ cells/well) into black 96-well plates and maintained to attach at 37°C in 5% CO_2_. After 24 h, cells were treated with 1.0 μM (~IC_50_) of the complex (**2**) for 1, 6, 12, and 24h. After that, cells were labeled with H_2_DCFDA solution at 5 μM in N,N-dimethylformamide (Sigma-Aldrich). After that, fluorescence intensity was measured in a Synergy H1 Fluorescence Spectrophotometer (BioTek^®^), at the excitation and emission wavelengths of 495 and 527 nm, respectively. Thereafter, ROS/Superoxide levels were measured using Cellular ROS/Superoxide Detection Assay Kit (Abcam^®^). Cells were seeded (1.5 × 10^4^ cells/well) into black 96-well plates and maintained to attach at 37°C in 5% CO_2_. After 24 h, cells were treated with complex (**2**) (0.25, 0.50, 1.0, and 1.5 μM) for 24 h. Negative control received the vehicle (DMSO 0.1% v/v), and positive control received pyocyanin (250 μM) as ROS inducer. Following this, cells were labeled with oxidative stress detection reagent (Green) for ROS detection and superoxide detection reagent (Orange) according to the manufacturer’s instruction. Fluorescence was measured in quadruplicates using a fluorescent microplate reader Synergy H1 (BioTek^®^) with standard fluorescein (Ex = 488 nm, Em = 520 nm) and rhodamine (Ex = 550 nm, Em = 610 nm) filter sets for ROS and superoxide determination, respectively. Protection assays using the antioxidant *N*-acetyl-l-cysteine (NAC) were also performed. In brief, the cells were treated with 5 mM NAC (Sigma-Aldrich) in association with complex (**2**) (0.50, 1.0, and 1.5 μM) for 24 h. Cells were labeled as described above, and the ROS levels were measured. In a new set of experiments, cells were treated with 5 mM NAC in association with cytotoxic concentrations of (**2**). After 24 h of treatment, the cell viability was determined by Alamar blue^®^ assay as described above.

### Assessment of DNA Damage by Comet Assay

The comet assay was performed to evaluate DNA damage following the method described by (37). Briefly, DU-145 and PNT-2 cells were seeded (1.0 × 10^5^ cells/well) in 24-well plates and maintained to attach at 37°C in 5% CO_2_ for 24 h. After, cells were treated with different concentrations of complex (**2**) for 24 h. DMSO (0.1% v/v) was used as the vehicle control and methyl methanesulfonate (150 μM, Sigma-Aldrich) as DNA damage inducer. Following 24 h of incubation, the cells were collected, and cell viability assay was performed using trypan blue dye. Analyzed samples showed more than 70% of viability (data not shown). Then, cells were mixed with UltraPure^™^ Low-Melting-Point Agarose (0.5%, Invitrogen^®^), and added on pre-coated slides with UltraPure^™^ Agarose (1.5%, Invitrogen^®^). After agarose solidification, the slides were immersed in a lysis solution (2.5 M NaCl, 100 mM EDTA, 10 mM Tris, 10% DMSO, 1% Triton X-100, pH 10.4) for 18 h at 4°C. After that, the slides were added with electrophoresis buffer (300 mM NaOH, 1 mM EDTA, pH > 13.4) to the electrophoresis chamber. The electrophoresis run was set up for 20 min of an electric field of 25 V and 300 mA (0.9 V/cm). Slides were neutralized (0.4 M Tris, pH 7.5, 4°C) and fixed in absolute ethanol for 5 min. Dried slides were stained with Gel Red^®^ (1:10,000, Uniscience^®^). The nucleoids were identified in a fluorescence microscope (AxioStar Plus, Carl Zeiss Axio Cam MRc-AxioVision 3.1) using a 516 to 560 nm filter, 590 nm filter barrier, in 20× objective with an integrated digital camera. For each treatment, 100 random nucleoids were analyzed, and the tail fluorescence was measured through the Comet Assay IV™ imaging system (Perceptive Instruments^©^, Bury St Edmunds, England).

### DNA Interactions Assay With pBR322 Plasmid

Plasmid pBR322 (38 µM, Sigma-Aldrich) was mixed with different concentrations (0.125–1.0 µM) of both complexes and incubated at 37°C for 18 h. Then, the loading buffer was added and the samples were analyzed by agarose gel electrophoresis for 90 min in 1% agarose gel using a Tris-acetate-EDTA (TAE) buffer (0.45 M Tris-HCl, 0.45 M acetic acid, 10 mM EDTA, pH 7.4), and ethidium bromide was employed for staining. Bands were visualized with a ChemiDoc MP imager (BioRad, USA).

### DNA Minor Groove Interaction Assay With Hoechst 33258

DNA minor groove interaction was assessed by examining the ability of the complexes to displace Hoechst 33258 (Thermo Fischer Scientific) from calf thymus DNA (ct-DNA) (Sigma-Aldrich). The ct-DNA solution was prepared by dilution of 2 mg of the ct-DNA in 1 ml of Tris-HCl buffer (4.5 mM Tris-HCl, 0.5 mM Tris-base, 50 mM NaCl, pH 7.4), and the concentration was determined by absorption spectrophotometric using the molar absorption coefficient 6600 mol^−1^ dm^3^ cm^−1^ at 260 nm (38). Thus, the ct-DNA (125 µM) was incubated with Hoechst (2.7 µM), and the extinction of the fluorescence intensity was monitored by the addition of different concentrations (0–125 µM) of both complexes in Tris-HCl containing 10% of DMSO. Fluorescence emission spectra were recorded from 300 to 500 nm after excitation wavelength of 343 nm, using an opaque 96-well plate, in a Synergy H1 Fluorescence Spectrophotometer (BioTek^®^), at 37°C.

### Gene Expression Analysis by qPCR Array

Briefly, DU-145 cells were seeded (1.0 × 10^6^ cells/well) into six-well plates and maintained to attach at 37°C in 5% CO_2_. After 24 h, the cells were treated with 1.0 μM of the complex (**2**). DMSO (0.1% v/v) was used as the vehicle control. After 12 h of incubation, total RNA was isolated from the cells using the RNeasy^®^ Mini kit (Qiagen, Germany) according to the manufacturer’s instructions. RNA was quantified by NanoDrop spectrophotometer (ND-2000; Thermo Fischer Scientific). The Agilent 2100 Bioanalyzer and Agilent RNA 6000 Nanochip kits were used to assess the total RNA quality as per manual instruction (Agilent, USA). The Agilent 2100 Expert software (Version B.02.07.SI532) was used with the Eukaryote Total RNA Nano assay on the Agilent 2100 bioanalyzer. No significant differences in RIN values were observed across the samples. Complementary DNA strands were synthesized using the RT^2^ First Strand kit (Qiagen, Germany), following the manufacturer’s instructions. Gene expression profiling was performed using the RT^2^ Profiler^™^ PCR Array Human Cancer Drug Targets (PAHS 507Z, Qiagen) by qPCR. The reactions were conducted in the Applied Biosystems 7500 PCR System (Applied Biosystems, USA) thermocycler. The plate was heated to 95°C for 10 min, followed by 40 cycles of 95°C for 15 s and 60°C for 1 min, followed by the dissociation curve. The expression profile of 84 key drug targets genes was analyzed. Fold changes amplification for targeted genes was normalized to the housekeeping genes *GAPDH* and *RPLP0* by the delta–delta cycle threshold method (ΔΔCt), and the cells treated with vehicle control (0.1% of DMSO v/v) was used as calibrator. The threshold cycle values of control wells were all within the ranges recommended by the PCR array user manual. Three independent biological replicates were performed. All experiments were performed in DNase/RNase free conditions. Genes were considered differentially regulated if the difference was ≥1.8-fold, which means that the gene expression in the compound-treated cells was at 1.8-fold that in the negative control-treated cells. Differences were considered significant at* p* ≤ 0.05. Statistical analysis of two groups was performed automatically according to the GeneGlobe Data Analysis Center (Qiagen, USA; https://geneglobe.qiagen.com/us/analyze).

### Protein Extraction and Western Blot

Briefly, DU-145 cells were seeded (1.0 × 10^6^ cells/well) into six-well plates and maintained to attach at 37°C in 5% CO_2_. After 24 h, cells were treated with 0.25, 0.50, 1.0, and 1.5 μM of complex (**2**). DMSO (0.1% v/v) was used as the vehicle control. After 24 h of incubation, cells were collected and suspended in Western blotting lysis buffer (20 mM Tris-HCl pH 7.4, 150 mM NaCl, 1 mM EDTA, and phosphatase and protease inhibitors). Cell lysates were centrifuged at 11,000*g* for 15 min at 4 °C, and supernatant was collected. Protein concentrations were quantified using the BCA Protein Assay Kit^®^ (Thermo Fischer Scientific), according to the manufacturer’s instructions. For immunoblotting, equal amounts (30 μg) of total protein were electrophoresed in a NuPAGE^®^ 4-12% Bis-Tris gel and transferred to an Invitrolon^™^ polyvinylidene difluoride (PVDF) membrane (Novex^®^, Thermo Fischer Scientific) at 100 V for 1 h. PVDF-membrane was washed two times (5 min each) with Tris-buffered saline plus Tween 20 (TBST, Sigma-Aldrich) and blocked in 5% non-fat milk in phosphate buffered saline with Tween^®^20 (PBST, Sigma-Aldrich) for 1 h at room temperature. After, the membranes were incubated 4 h at 18°C with primary antibodies against β-actin (ab8226, Abcam, 1:1000), PARP1 (ab74290, Abcam, 1:1000), cleaved PARP1 (ab32064, Abcam, 1:1000), cleaved caspase-3 (ab2302, Abcam, 1:1000), cleaved caspase-8 (MA5-15054, Thermo Fischer Scientific, 1:500), cleaved caspase-9 (ab2324, Abcam, 1:1000), and aurora B (ab2254, Abcam, 1:1000), previously diluted in blocking solution to appropriate concentrations. After removing unbound primary antibodies, membranes were washed four times (7.5 min each) with TBST buffer (100 mM Tris- HCl, 300 mM NaCl, 1% Tween 20) and incubated with the respective secondary goat anti-mouse HRP antibody (1:3000 diluted in 1% non-fat milk in TBST; NA931V, GE Healthcare) for 1 h at room temperature. Membranes were washed additional eight times (7.5 min. each) with TBST before performing the enhanced chemiluminescence step using ChemiDoc^™^ MP Image System (Bio-Rad, USA). Digital images were acquired using a software Scion Imaging (Scion Corporation, USA). The protein β-actin was used for sample loading normalization.

### Statistical Analysis

Data are presented as mean ± S.E.M. or as IC_50_ values with 95% confidence intervals (CI 95%) obtained by nonlinear regression. Differences between experimental groups were compared using analysis of variance (ANOVA) followed by the Dunnet’s or Tukey’s test (*p* < 0.05). All statistical analyses were performed using GraphPad Prism 8 for MacOS X (Intuitive Software for Science, USA).

## Results

### Synthesis of Novel Lapachol-Containing Ruthenium Complex

Two novel ruthenium complexes (**1** and **2**), with the general structures represented in **Figure 1**, were synthesized from the reaction of the precursor complex *cis*-[RuCl_2_(dppm)(N-N)], where N-N = 2,2'-bipyridine (bipy) or 1,10-phenanthroline (phen) and dppm = 1,1′-*bis*(diphenylphosphine)methane.

The structures for the complexes were confirmed by analytical and spectroscopic procedures described in *Material and Methods* section. The establishment of monocationic complexes was verified by molar conductivity measurements with a conductivity range between 17.22 and 59 Ω^−1^ cm^2^ mol^−1^, which is consistent with the proposed structures. The elemental analyses of the complexes are coherent with their proposed formulas.

Infrared spectra of the complexes (**1**) and (**2**) confirmed the coordination of the Lap ligand. The band assigned to the *v*(O-H) stretching vibration of Lap was not observed in all complexes, indicating the deprotonation. The *v*C_1_ = O and *v*C_2_−O of free Lap present values of 1,643 and 1,050 cm^−1^, respectively. In the complex (**1**) the *v*C_1_=O stretching vibration was shifted to lower frequencies, 1546 cm^1^, and the *v*C_2_−O shifted to 1103 cm^−1^ (**Supplementary Figures 1** and **2**). These results are in agreement with the change in electronic density after the coordination of the Lap to ruthenium that occurred in a bidentate mode by the oxygens atoms (16). Also, characteristic bands of a $PF_{6}^{-}$ counterion were observed at 840 and 555 cm^−1^. The same comportment was observed for complex (**2**).

The UV-vis spectra of both complexes showed bands around 268 and 293 nm characteristic of intraligand transitions (π → π*) of the bipy, phen, dppm, and Lap ligands. Also, bands around 350 to 600 nm was observed and can be assigned to metal-to-ligands charge transfer (MLCT) and, n → π* transitions of quinone carbonyl groups (8, 39, 40). The cyclic voltammograms showed a process related to Ru^II^/Ru^III^ oxidation at 1,188 and 1,176 mV, for complex (**1**) and (**2**), respectively, which are higher than the precursor complexes (0.1–0.6 V) (41). This observation indicates the higher stability of the complexes after the lapachol coordination to the metal.

In the ^31^P{^1^H} NMR spectra of complexes (**1**) and (**2**), it was observed a typical AB spin system with chemical shifts at 18.2/6.2 and 14.7/5.8 ppm for complex (**1**) and 18.3/7.1 and 14.9/6.5 ppm, for complex (**2**), indicating the nonequivalence of the phosphorus atoms. The duplication of phosphorus signals in the ^31^P{^1^H} NMR spectra are related to the presence of isomers in solution. In one isomer the phosphorus atom from the dppm ligand is *trans* to oxygen in the carbonyl of the lapachol and in another, the phosphorus atom is *trans* to the oxygen of enol (**Supplementary Figures 3** and **4**). The same behavior has been observed for others Ru(II) complexes with naphtoquinones as ligands (23, 40).

The ^1^H NMR spectra of both complexes agree with the proposed structures of complexes. For complex (**1**), in the range 8.7 to 6.6 ppm it was observed multiplets referent to 32H atoms of the bipy, dppm and Lap. There were observed multiplets at 5.5, 4.8, and 3.1 ppm of CH and CH_2_ groups of the aliphatic chains of Lap and dppm. Also, 1.5 to 1.4 ppm was observed signals attributed to the two CH_3_ groups of Lap (**Supplementary Figures 5** and **6**).

The ^13^C NMR spectra of both complexes displayed signals characteristic of C1 = O and C2−O groups of Lap around 196 and 181 ppm. These signals were observed around 181 and 155 ppm in Lap free (**Supplementary Figures 7** and **8**). The displacement observed indicates the change in electronic density after the coordination of the Lap to ruthenium. It is noteworthy the stability of the complexes was evaluated before conducting the cell experiments. The stability was studied using UV-vis and cyclic voltammograms experiments where both complexes demonstrated great stability in DMSO and DMSO/Tris-HCl buffer (pH 7.4) for a period of at least 72 h (**Supplementary Figures 9** and **10**).

### Partition Coefficient (log P) Study

Lipophilicity is an essential physicochemical feature that predicts the pharmacokinetic/pharmacodynamic profile of new drug candidates drugs (42). The classical shake-flask method is widely recognized to determine the partition coefficient experimentally (43). The log P(o/w) values found were positive and ranged from 0.617 ± 0.014 to complex (**1**) and 0.839 ± 0.007 to complex (**2**). The phenanthroline ligand might have granted higher lipophilicity to complex (**2**) which could be related to its cytotoxicity described below.

### Lapachol-Containing Ruthenium Complexes Display Potent Cytotoxicity Against a Panel of Cancer Cell Lines

The potential of the complexes to disturb the viability of different histological types of cancer cell lines (A-375, A549, Caco-2, DU-145, HepG2, PC-3, and MDA-MB-231) and two non-cancer cells (FGH and PNT-2) was assessed by Alamar blue^®^ assay. **Table 1** shows that both complexes presented enhanced cytotoxicity to different cancer cells and more potent activity than metal-free lapachol. The synthesized lapachol-containing ruthenium complexes exhibited remarkable cytotoxic properties against prostate cancer cells DU-145 and PC-3. Complex (**1**) presented IC_50_ values of 0.9 to 3.3 μM and complex (**2**) presented IC_50_ values of 0.8 to 2.6 μM for cancer cell lines DU-145 and A549, respectively. The complexes also displayed higher cytotoxic activity than cisplatin (Fauldcispla^®^) and doxorubicin (Fauldoxo^®^). Doxorubicin presented IC_50_ values ranging from 1.9 to 3.7 μM for cancer cell lines A549 and A-375, respectively. Cisplatin presented IC_50_ values varying from 10.8 to 24.3 μM for cancer cell lines PC-3 and DU-145, respectively. The precursors *cis*-[RuCl_2_(dppm)(bipy)] and *cis*-[RuCl_2_(dppm)(phen)]) had been previously tested and exhibited only weak cytotoxicity (IC_50_ > 100 μM) (23) and were not tested in the present work. The IC_50_ values for non-cancer cells were 3.1 and 5.9 μM for the complex (**1**), and 2.5 and 6.8 μM for the complex (**2**) for FGH and PNT-2 cells, respectively. In addition, the IC_50_ value for non-cancer cells was 2.6 and 3.2 μM for doxorubicin and 11.4 and 9.5 μM for cisplatin for FGH and PNT-2 cells, respectively.

**Table 1:** Cytotoxic activity of lapachol-containing ruthenium complexes.

	IC_50_ [µM]				
	DXR	CIS	(1)	(2)	Lp
***Cancer cells***					
**A-375**	3.7 ± 0.6	16.5 ± 2.3	2.5 ± 0.6	0.9 ± 0.3	>100.0
**A549**	1.9 ± 0.4	11.9 ± 0.6	3.3 ± 0.4	2.6 ± 0.1	>100.0
**Caco-2**	2.1 ± 0.8	17.4 ± 0.3	2.0 ± 0.6	1.2 ± 0.3	>100.0
**DU-145**	2.3 ± 0.5	24.3 ± 1.3	0.9 ± 0.2	0.8 ± 0.1	>100.0
**HepG2**	2.4 ± 0.8	14.1 ± 0.5	1.7 ± 0.5	1.5 ± 0.2	>100.0
**MDA-MB-231**	2.1 ± 0.7	22.1 ± 1.7	2.1 ± 0.4	2.0 ± 0.0	>100.0
**PC-3**	2.6 ± 0.3	10.8 ± 1.1	1.3 ± 0.4	1.1 ± 0.3	>100.0
***Non-cancer cells***					
**FGH**	2.6 ± 0.6	11.4 ± 0.5	3.1 ± 0.2	2.5 ± 0.3	>100.0
**PNT-2**	3.2 ± 0.5	9.5 ± 0.3	5.9 ± 2.1	6.8 ± 1.5	>100.0

Data are presented as the means ± S.E.M. of IC_50_ values in μM obtained by nonlinear regression from at the least three independent experiments performed in triplicate, measured by Alamar blue^®^ assay after 24-h incubation. Cancer cells: A-375 (human malignant melanoma); A549 (human lung carcinoma); Caco-2 (human colorectal adenocarcinoma); DU-145 (human prostate adenocarcinoma); HepG2 (human hepatocellular carcinoma); MDA-MB-231 (human breast adenocarcinoma) and PC-3 (human prostate adenocarcinoma). Non-cancer cells: FGH (human mouth fibroblast) and PNT-2 (human prostate epithelial cells). Cisplatin (CIS), doxorubicin (DXR) and lapachol (Lp) were used as controls.

The selectivity index (SI) measured for each complex has been presented in **Supplementary Table 1**. The SI was calculated using: SI = IC_50_ [non-cancer cells]/IC_50_ [cancer cells]. The SI is a clear manner to estimate the therapeutic index of a drug and to identify safe drug candidates for further investigations (44). Complexes (**1**) and (**2**) were more cytotoxic and selective to prostate cancer cells DU-145. In fact, complex (**2**) showed higher selectivity for prostate cancer cells (SI = 8.5, for DU-145 and SI = 6.2, for PC-3) when compared to prostate non-cancer cells. Furthermore, both complexes exhibit SI greater than displayed by the reference drugs cisplatin and doxorubicin. In a further round of experiments, the DU-145 was used as a cellular model, since displayed higher sensibility to treatments.

**Figure 2:**
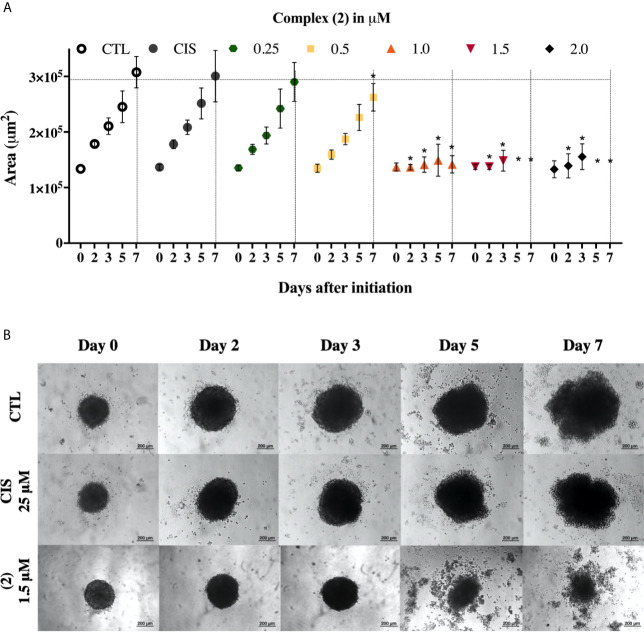
Effect of the complex (**2**) in 3D *in vitro* model of multicellular tumor spheroids of DU-145 cells. **(A)** Routine monitoring of spheroid growth of DU-145 MCTSs treated with 0.25, 0.5, 1.0, 1.5, and 2.0 μM of complex (**2**) without medium renewal followed by 50% medium exchange every 48 h for further culturing. Every feeding step results in a consecutive 1:2 dilution of the treatment. Graph represents spheroid volume growth kinetics of DU-145 MCTSs in liquid overlay culture. **(B)** DU-145 MCTSs were examined using inverted microscope (amplification, 200×), scale bar = 200 μm. The negative control (CTL) was treated with the vehicle (0.1% DMSO) used for diluting the complex. Cisplatin (CIS 25 μM) were used as the positive controls. Data are presented as the mean ± S.E.M. of three independent experiments performed in triplicate. *p < 0.05 compared with the control by ANOVA followed by Dunnet’s test.

We also evaluate the complexes toward 3D cell cultures based on DU-145 cells. Interestingly, the prostate multicellular tumor spheroids (MCTSs) undergo alterations in their structure, indicating an effective permeability and, consequently cytotoxicity. The treatment of DU-145 MCTSs with complex (**2**) caused a decrease in the total spheroid area observed over time, which possibly resulted from cell apoptosis (**Figure 2A**). Subsequently, the treatment perturbed the cell aggregations, resulting in the presence of cell debris after 5 days, indicating an effective drug permeability and cytotoxicity in the 3D cultures (**Figure 2B**). The IC_50_ of the complexes (**1**) and (**2**) against the DU-145 MCTSs, were 2.4 and 1.9 μM, respectively, while cisplatin presented IC_50_ of 62.6 μM. The complex (**2**) was more potent than cisplatin at the least 32-fold, showing that its cytotoxic activity is effective in the tumor spheroids of DU-145 cells.

### Lapachol-Containing Ruthenium Complexes Inhibits Cell Proliferation and Induces Cell Cycle G2/M Arrest in DU-145 Cells

**Figure 3:**
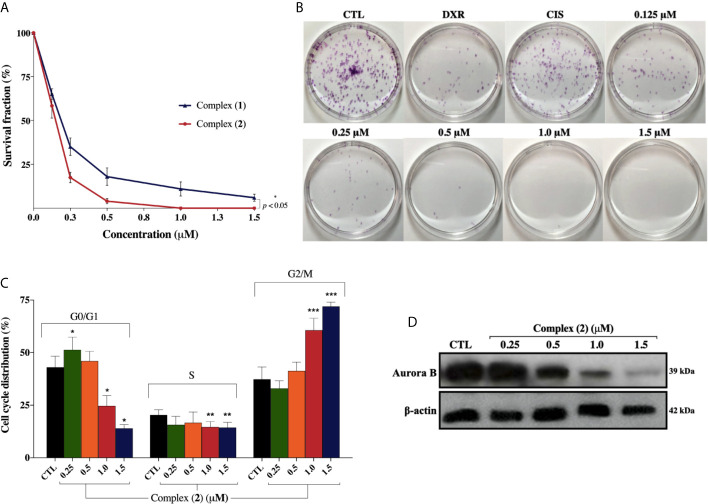
Effect of lapachol-containing ruthenium complexes in DU-145 cell proliferation and cell cycle kinetics. **(A)** The survival fractions after 24h treatment with 0.125; 0.25; 0.5; 1.0, and 1.5 μM of complexes (**1**) and (**2**) against DU-145 cells. **(B)** Representative colony formation images of DU-145 cells after treatment with different concentrations of complex (**2**), DXR (2.5 μM) and CIS (25 μM). **(C)** Cell cycle distribution of DU-145 cells after treatment with complex (**2**) for 24 h. *, **, and *** indicate significant difference compared to the CTL in phase G0/G1, S, and G2/M, respectively. **(D)** Western blot analysis showing the expression status of Aurora-B in prostate adenocarcinoma cells after treatment with complex (**2**) for 24 h. The densitometric analysis of gray bands is shown in **Supplementary Figure 11**. The negative control (CTL) was treated with the vehicle (DMSO 0.1% v/v) used for diluting the complex. Data are presented as the mean ± S.E.M. of three independent experiments performed in duplicate. Two-way ANOVA followed by Dunnett’s post-test (*p *< 0.05).

Both complexes inhibited colony formation at low cell density in a concentration-dependent manner, with partial inhibition at 0.125 μM (**Figure 3A**). Mainly, the inhibitory effect of the complex (**2**) was greater than that of the complex (**1**) at the same concentrations. At concentrations of 0.125, 0.25, 0.5, 1.0, and 1.5 μM, the complex (**1**) reduced colony formation, presenting survival fractions (SF) of 65.1%, 35.1%, 18.3%, 9.0%, and 4.3%, respectively. Complex (**2**), at the same concentrations, presented SF of 58.0%, 17.7%, 4.3%, 0%, and 0%, respectively. No significant (*p* > 0.05) decrease in the number of colonies was observed in control (DMSO 0.1% v/v). Doxorubicin at 2.5 μM and cisplatin at 25 μM presented SF of 18.7% and 69.1%, respectively. At concentrations of 1.0 and 1.5 μM of complex (**2**), no colonies were visible at the plates (**Figure 3B**). The plating efficiency (PE) of untreated cells was higher than 76% for all replicates. Our findings revealed that both the complexes and doxorubicin were efficient in inhibiting cell proliferation after 24 h of treatment. Nonetheless, the same efficacy was not observed with cisplatin which presented SF of 69.1%, suggesting that, in 24 h, the complexes were more efficient than the metallodrug. Also, cell cycle analyses were carried out to investigate the relationship between inhibition of cell proliferation found in colony-formation assay and cell cycle arrest. The complex (**2**) increased cell number at the G2/M phase after 24 h treatment with increasing concentration, followed by decreased cell number at G0/G1 and S phases in DU-145 cells (**Figure 3C**).

To further evaluate the antiproliferative activity of complex (**2**), its effect on the expression of Aurora-B kinase was examined, since it is a mitotic checkpoint that performs a critical role during mitosis, guaranteeing correct chromosome segregation (45). Some studies also indicated that Aurora-B is overexpressed in human prostate adenocarcinoma and the inhibition of its kinase activity decreases considerably the cell proliferation (46). As shown in **Figure 3D**, we found that Aurora‐B levels were downregulated after treatment of increasing concentrations of complex (**2**). Similar results were observed at the mRNA level as demonstrated on **Table 2**. Overall, these data indicated that the antiproliferative action of complex (**2**) in DU-145 prostate cells is associated with Aurora-B downregulation and cell cycle arrest in G2/M phase.

**Table 2:** The effect of lapachol-containing ruthenium complex (**2**) on gene expression of DU-145 cells.

Gene symbol	Description	Fold change	*P* value
*AKT1*	*AKT* serine/threonine kinase 1	−2.6	0.0008
*AKT2*	*AKT* serine/threonine kinase 2	−2.6	0.0026
*AURKA*	Aurora kinase A	−5.3	0.0003
*AURKB*	Aurora kinase B	−11.1	0.0005
*AURKC*	Aurora kinase C	−3.0	0.0027
*BCL2*	*BCL2* apoptosis regulator	−4.1	0.0053
*BIRC5*	Baculoviral IAP repeat containing 5	−6.4	0.0031
*CDC25A*	Cell division cycle 25A	−2.6	0.0042
*CDK1*	Cyclin dependent kinase 1	−4.5	0.0029
*CDK2*	Cyclin dependent kinase 2	−2.7	0.0034
*CDK4*	Cyclin dependent kinase 4	−4.6	0.0018
*CDK5*	Cyclin dependent kinase 5	−3.2	0.0003
*EGFR*	Epidermal growth factor receptor	−4.1	0.0008
*ERBB2*	Erb-b2 receptor tyrosine kinase 2	−2.4	0.0001
*GSTP1*	Glutathione S-transferase PI 1	−2.9	0.0005
*HDAC8*	Histone deacetylase 8	−1.8	0.0002
*IGF1R*	Insulin-like growth factor 1 receptor	−2.7	0.0012
*MDM4*	*MDM4* regulator of P53	2.8	0.0067
*MTOR*	Mechanistic target of rapamycin kinase	−3.8	0.0016
*PARP1*	Poly(ADP-ribose) polymerase 1	−2.7	0.0004
*PARP2*	Poly(ADP-ribose) polymerase 2	−3.0	0.0087
*PDGFRB*	Platelet-derived growth factor receptor beta	−2.0	0.0025
*PIK3CA*	Phosphatidylinositol-4,5-bisphosphate 3-kinase alpha	−2.7	0.0032
*PTGS2*	Prostaglandin-endoperoxide synthase 2	2.1	0.0006
*TOP2A*	DNA topoisomerase II alpha	−2.1	0.0006
*TOP2B*	DNA topoisomerase II beta	−2.8	0.0002

DU-145 cells were treated with 1.0 µM of complex (**2**) for 12 h. The negative control was treated with the vehicle (DMSO 0.1% v/v) used for diluting the compound. After treatment, total RNA was isolated and reverse transcribed. Gene expression was detected using the 96-well plate Human Cancer Drug Targets RT^2^ Profiler PCR Array^®^. GAPDH and RPLP0 genes were used as endogenous gene for normalization. Panel shows selected genes revealing more than 1.8-fold regulation and P value < 0.05. Analysis was performed using the delta–delta cycle threshold method (ΔΔCt). Changes in the gene expression were illustrated as a fold increase or decrease.

### Lapachol-Containing Ruthenium Complexes Triggers Caspase-Mediated Apoptosis of DU-145 Cells

**Figure 4:**
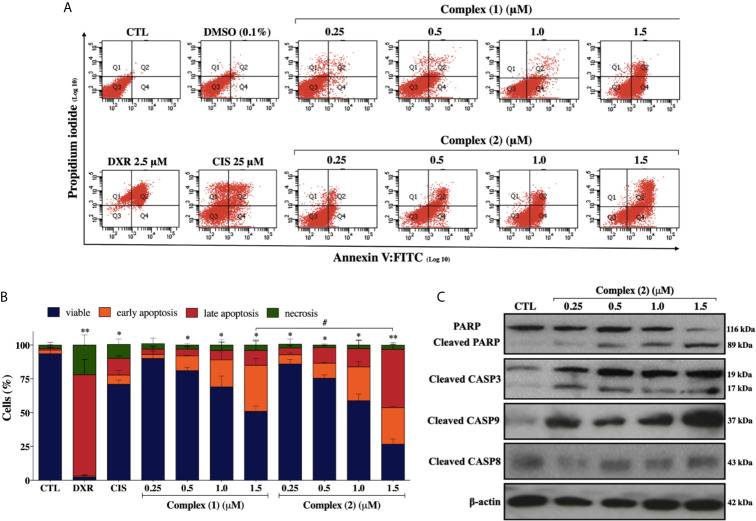
Lapachol-containing ruthenium complexes induces apoptosis in DU-145 cells. **(A)** Representative flow cytometry dot plots show the presence of cells on viable (Q3), early apoptotic (Q4), late apoptotic (Q2) and necrotic (Q1) stages and, **(B)** showed the summary data determined by flow cytometry using annexin V-FITC/PI staining. Data are presented as the means ± S.E.M. of three independent experiments performed in triplicate. Ten thousand events were evaluated per experiment, and cellular debris was omitted from the analysis. **(C)** Western blot analysis showing the expression and activation status of cleaved PARP as well as cleaved caspase-3 and -9 levels in prostate adenocarcinoma cells after treatment with complex (**2**) for 24 h. The densitometric analysis of gray bands is shown in **Supplementary Figure 11**. The negative control (CTL) was treated with the vehicle (0.1% DMSO) used for diluting the tested compound and, doxorubicin (DXR 2.5 μM) and cisplatin (CIS 25 μM) were used as drug control. Two-way ANOVA followed by Dunnett’s post-test where, *p < 0.05 *vs.* apoptotic cells in CTL; **p < 0.01 *vs.* apoptotic cells in CTL; ^#^ *p* < 0.05 apoptotic cells after 1.5 μM of complex (**1**) *vs.* apoptotic cells after 1.5 μM of complex (**2**).

To assess whether the cytotoxicity of the complexes is associated with cell death, we performed Annexin-V/PI double staining. The flow cytometry analysis showed that both complexes increased the number of cells in the early and late apoptosis stages (Q2 and Q4 quadrants) in a concentration-dependently manner (**Figure 4A**). For the complex (**2**), the population of apoptotic cells have increased remarkably in a concentration-dependent manner (5.9% for control group, 11.8% for 0.25 μM, 22.7% for 0.5 μM, 41.4% for 1.0 μM, and 70.2% for 1.5 μM) (**Figure 4B**). The complexes did not induce a significant increase of necrotic cells after the treatments. Cisplatin treatment of the DU-145 cells, at the fixed concentration of 25 μM (~IC_50_) showed 19.5% of apoptotic cells. Therefore, a 100-fold lower concentration of complex (**2**) was required for the same effect of cisplatin on DU-145 cells. To further determine whether extrinsic or intrinsic pathway the complex (**2**) induced apoptosis, we evaluated the expression of downstream apoptotic proteins by western blot. As shown in **Figure 4C**, there was an increase in the activation of cleaved caspase-3, -9, and PARP. Taken together, these data indicated that complex (**2**) induced apoptosis by activating the intrinsic pathway. Consequently, complex (**2**) was chosen for comprehensive studies of the mechanism of cell death.

### Lapachol-Containing Ruthenium Complex (2) Increase ROS Levels and Induce DNA Damage on DU-145 Cells

**Figure 5:**
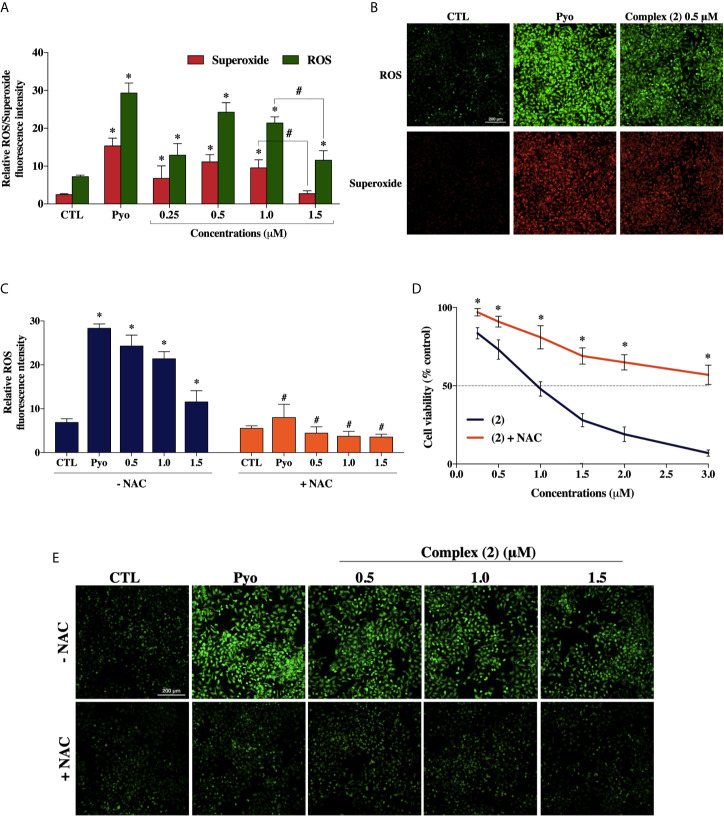
Effect of the ruthenium complex (**2**) in the levels of reactive oxygen species (ROS) of DU-145 cells. **(A)** Quantitative analysis of relative ROS/Superoxide fluorescence emission intensity 24 h after incubation with different concentrations of complex (**2**). **(B)** Cells labeled with the ROS-sensitive fluorescent dyes using the Cellular ROS/Superoxide Detection Assay after exposure at 0.5 μM of the complex (**2**). **(C)** ROS levels of DU-145 cells after 24 h treatment with complex (**2**) at 0.5, 1.0, and 1.5 μM with or without the antioxidant NAC (5 mM). **(D)** Cell viability of DU-145 cells assessed by Alamar blue^®^ assay after 24 h incubation with different concentrations of (**2**) with or without the antioxidant NAC (5 mM). **(E)** ROS scavenger NAC reduces the generation of intracellular ROS induced by complex (**2**). All fluorescence images were acquired using INCell Analyzer 2000 system at a total magnification of 200× (scale bar = 200 μm). The negative control (CTL) was treated with the vehicle (0.1% DMSO) used for diluting the tested compounds. Pyocyanin (Pyo, 250 μM) was used as the positive control. Data are presented as the mean ± S.E.M. of three independent experiments performed in triplicate or quadruplicate. *p < 0.05 compared with the control by ANOVA followed Dunnet’s test. ^#^ *p* < 0.05 compared with the respective treatment without NAC by ANOVA followed Dunnet’s test.

To explore whether ROS are involved with the cytotoxicity of the complex (**2**), we used the Cellular ROS/Superoxide Detection Assay (Abcam, UK). Previously, the total ROS levels were assessed after 1, 6, 12, and 24 h of incubation with 1.0 μM of complex (**2**) (~IC_50_) using H_2_DCFDA as a fluorescent dye to determine which time would be more appropriate for a ROS/Superoxide analysis. The treatment of 24 h showed the highest increase of ROS (**Supplementary Figure 12**), so the ROS/Superoxide detection was assessed after 24 h of exposure with several concentrations of complex (**2**). We determined that total ROS levels in DU-145 cells were significantly higher after the treatment (**Figure 5A**), suggesting the complex (**2**) exposure induced an oxidative stress response in the prostate tumor cells. It is demonstrably in **Figure 5B**, that the treatment induced ROS/Superoxide accumulation in cells with the highest amount of ROS produced after exposure to 0.5 μM of the complex (**2**). Moreover, at 1.5 μM there was observed a decrease of ROS/Superoxide levels, probably because increasing concentrations promotes cell death.

Besides, to verify the role of ROS in the trigger of cytotoxicity by complex (**2**), we used the ROS-scavenger NAC, an antioxidant that has been proven to display cytoprotective effects against ROS-induced cytotoxicity (47). The cotreatment with ROS scavenger inhibited the formation of intracellular ROS induced by complex (**2**) (**Figures 5C, E**) and, as expected, prevented the reduction of cell viability, as assessed by Alamar blue^®^ assay (**Figure 5D**).

**Figure 6:**
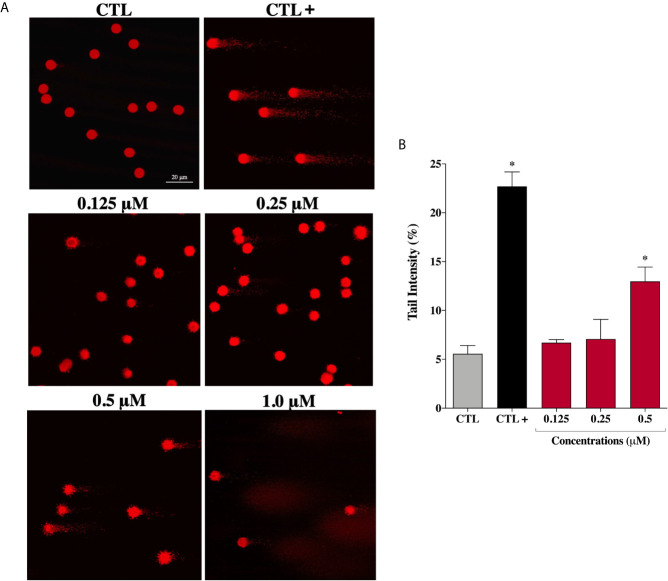
Genotoxic effect of ruthenium complex (**2**) in DU-145 cells. **(A)** Representative image samples obtained by application of alkaline version of comet assay on DU-145 cells treated with complex (**2**) at 0.125, 0.25, 0.5, and 1.0 μM for 24 h. **(B)** Bar graph represents the tail intensity (%), which is directly correlated to damage DNA. The images were acquired with fluorescence microscopy at a total magnification of 400× (scale bar = 20 μm) and correspond to a representative assay from three independent experiments. The negative control (CTL) was treated with the vehicle (0.1% DMSO) used for diluting the tested compound and the positive control (CTL+) was treated with methyl methanesulfonate (150 μM). Data are presented as the mean ± S.E.M. of three independent experiments. *p < 0.05 compared with the control by ANOVA followed Dunnet’s test.

Considering that ROS are known to trigger DNA damage, the genotoxicity of the complex (**2**) on prostate cells were investigated by comet assay, a sensitive method for DNA strand break revelation. As shown in **Figure 6A**, in the control group, the nucleoids of DU-145 cells are round-shaped containing the undamaged DNA. However, after 24 h treatment, DU-145 cells exhibited well-formed comet tails by denatured DNA fragments migrating out of the cell nucleus during electrophoresis, which indicate the existence of DNA damage. We observed a significant DNA damage increase in DU-145 nucleoids treated with complex (**2**) at 0.5 μM (**Figure 6B**). Attempts to measure DNA damage after 24 h treatment with 1.0 μM of complex (**2**) gave inconsistent results with presence of nondetectable cell nuclei due the extensive cell death and DNA fragmentation during processing of DU-145 cells.

However, under the same experimental conditions, prostate normal cells showed no DNA damage after treatment with complex (**2**) at the same concentrations. Only at the maximum tested concentration of 6.0 μM (~IC_50_) an increase in DNA damage was detected on PNT-2 cells (**Supplementary Figure 13**). These results indicate that complex (**2**) can enhance intracellular oxidative stress suggesting that apoptosis may be the principal mechanism of cell death triggered by ROS-mediated DNA damage.

### Lapachol-Containing Ruthenium Complexes Interact With DNA

**Figure 7:**
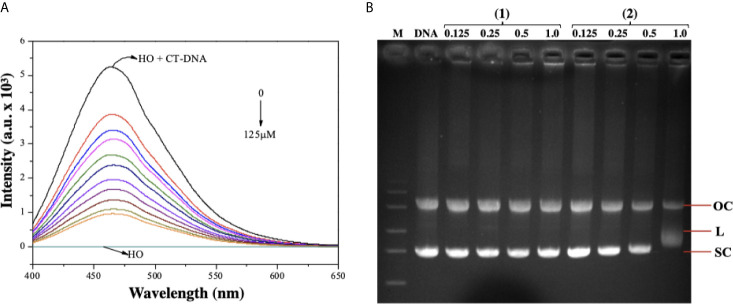
Interaction of ruthenium-containing ruthenium complexes with DNA molecule. **(A)** Emission spectra of Hoechst (HO) (2.7 µM, λ_ex_ = 343 nm), CT-DNA (125 µM) in presence of the complex (2) in different concentrations (0 - 125 µM). **(B)** Agarose gel electrophoresis image of pBR322 plasmid DNA incubated with complexes (**1**) and (**2**). The pBR322 presents three forms, OC (open circular), L (linear) and SC (supercoiled). Lane 1 (M): molecular weight marker; lane 2 (DNA): pure pBR322 with DMSO; lanes 3–6 [complex (**1**)] and lanes 7-10 [complex (**2**)].

As complex (**2**) was able to cause DNA damage, we studied the capability of these lapachol-containing ruthenium complexes to interact with ct-DNA by fluorescence and gel electrophoresis methods. DNA minor groove interaction was assessed by investigating the ability of the complexes to replace Hoechst 33258 from ct-DNA. To achieve this, the complexes were added to a DNA-Hoechst 33258 mixture to assess if the complexes compete with Hoechst and interact with DNA. If the complexes displace Hoechst from DNA, the fluorescence intensity of Hoechst decreases. Indeed, complex (**2**) could decrease the fluorescence of the Hoechst-DNA system by enhancing the concentration, indicating that it interacts with DNA by the minor groove and competes with Hoechst, removing it from DNA structure, thereby decreasing the fluorescence of the system (**Figure 7A**). Similar behavior was observed in complex (**1**) (**Supplementary Figure 14**). In addition, complex (**2**) was able of influencing the mobility of the bands of pBR322 plasmid DNA in electrophoresis in agarose gel. Modification of the DNA structure causes interference in the migration of supercoiled DNA and a slight increase in the mobility of open circular DNA where both forms comigrate. The complex (**2**) was found to exhibit nuclease activity followed by conversion of supercoiled (SC) into linear (L), proposing single-strand DNA cleavage at 1.0 μM (**Figure 7B**). Taken together, these findings suggest indicating that complex (**2**) directly interacts with DNA.

### Lapachol-Containing Ruthenium Complex (2) Alters Gene Expression in DU-145 Cells

The expression of several genes associated with important cancer drug targets, such as cell proliferation, apoptosis, DNA damage repair, and epigenetics, was investigated in prostate cancer cells treated with 1.0 µM of complex (**2**) for 12 h by qPCR using a 96-well plate Human Cancer Drug Targets RT^2^ Profiler PCR Array^®^. To screen out inaccurate results a 1.8-fold change in gene expression was applied as the cutoff point. The assessment shows a significant change in 26 genes (30.9%) in the treated DU-145 cells compared to the untreated cells. A total of two genes were upregulated while 24 genes were downregulated (**Table 2**). The results demonstrate alterations in the expression of several genes involved in cellular pathways, especially apoptosis, cell cycle, and cell signaling. Among them, the genes *BCL2* (-4.1-fold), *BIRC5* (-6.4-fold), *CDK1* (-4.5-fold), *CDK4* (-4.6-fold), *AURKA* (-5.3-fold), *AURKB* (-11.1-fold), and *AURKC* (-3.0-fold) were downregulated in the cells treated with complex (**2**), while the genes *MDM4* (2.8-fold) and *PTGS2* (2.1-fold) were upregulated.

Curiously, the complex (**2**) decreased markedly the expression of Aurora kinase genes, especially *AURKB* (-11.1-fold). The inhibition of this class of serine/threonine kinases leads to stop cytokinesis and promote cell growth repression. This effect may indicate that the complex (**2**) inhibits the progression of cell cycle, resulting in its antiproliferative effect demonstrated by inhibiting the clonogenicity of DU-145 cells.

## Discussion

Lapachol is a well-characterized antitumor agent, capable of exerting significant growth inhibitory effects in several cancer models through ROS-mediated cytotoxicity (48). A broad range of biological and pharmacological activities has been discovered for lapachol and its derivatives highlighting its antitumor activity, firstly described in the early 1970s (49, 50). Nevertheless, this class of natural compounds had never been employed, from our best knowledge, as a ligand for the composition of ruthenium(II)/phosphine complexes until the first studies of our research group (8, 51). Herein, novel lapachol-containing ruthenium(II)/phosphine complexes were synthesized, characterized, and evaluated for their anticancer potential.

Ruthenium complexes have been extensively reported as potent cytotoxic and anti-metastatic agents in different cancer cells (52, 53); however, the incorporation of bioactive ligands enhances the biological activity of the metal complex formed. Ruthenium(II) complexes designed in the present study were tested against several cancer cells, presenting important cytotoxic activity. Other recent studies have shown that complexes of ruthenium with different phosphine ligands can induce cytotoxic activity against cancer cell lines (54–56). Lapachol and its derivatives showed antiproliferative effects in different histological types of cells, including ovary, colon, lung, breast, leukemia, esophageal, cervical, melanoma, and prostate (7, 57–63). In this study, both lapachol-containing ruthenium(II) complexes displayed cytotoxic activity up to 100-fold higher than metal-free lapachol. Moreover, the complexes demonstrated selectivity for cancer cells greater than exhibited by the drugs cisplatin and doxorubicin, already used in the clinics for decades.

In 3D cell culture experiments, both complexes were also more potent in inducing cytotoxicity than cisplatin. Comparable with solid tumors *in vivo*, the hypoxic regions inside the spheroids decreases the velocity of cell division and diminishes the activity of chemotherapeutic drugs such as cisplatin (64). In our outcomes, the treatment with complex (**2**) provoked alterations in the shape of the spheroid, such as loss of circularity and consequent disaggregation. These findings are consistent with an effective permeability of the complex inside the spheroids.

Regarding their mechanism of action, complex (**2**) induce caspase-dependent apoptosis on DU-145 cells as observed by externalization of phosphatidylserine, DNA fragmentation, and caspase-3 and -9 activation. Besides, our data showed that complex (**2**) could suppress cell proliferation that was accompanied by an accumulation of cells in the G2/M phase, which is consistent with the downregulation of Aurora-B kinase in prostate cancer cells. The G2/M checkpoint blocks cells from entering mitosis with genomic DNA damage, granting an opportunity for repair or stopping the proliferation of damaged cells (65). According to the analysis, complex (**2**) increased the proportion in the G2/M phase and decreased the cell proportion in G0/G1 and S phases in DU-145 cells. Further, gene and protein expression analysis showed that complex (**2**) led to a decrease in the expression of the Aurora kinase family, an important group of enzymes that execute an essential function in cell cycle progression especially at the mitotic stage. In the current study, we show that the treatment with complex (**2**) decreased the expression of Aurora-B, followed by antiproliferative effects in DU-145 cells, such as loss of its clonogenicity. Studies also revealed that Aurora-B regulates the G2/M phase transition through several key factors at the transcriptional level (66, 67). Overexpression of Aurora-B has been observed in various cancers and has been associated with a worse prognostic for cancer patients (68). Besides, Aurora-B expression directly correlates with Gleason grade, an important prognostic factor in prostate cancer (46).

Some studies propose that Aurora-B expression is inhibited by proteins of DNA repair system (69–71). The repair of DNA damage is started by sensory proteins that accumulate at the sites of damage. This accumulation activates a cascade of phosphorylation that modifies the chromatin and allows access to DNA repair factors. The non-homologous end joining (NHEJ) factor Ku70 is phosphorylated in serine 155 in response to the DNA damage (72). When activated, Ku70 S155 interacts with Aurora B inhibiting its kinase activity, providing time to complete DNA repair. Taken together, these shreds of evidence suggest that complex (**2**) induces oxidative DNA damage and inhibits Aurora-B on DU-145 cells. The principal mechanism of action is the ability of this complex to increase the generation of ROS, thereby being responsible for its ability to cause DNA damage and trigger apoptosis through the intrinsic pathway.

As mentioned above, lapachol induces the generation of ROS, which damages DNA and afterward promotes apoptosis. It is widely known that ruthenium(II) complexes could lead to apoptosis through ROS-mediated pathway in cancer cells (73, 74). ROS are recognized as critical upstream molecules in the regulation of apoptosis and cancer initiation. Basal levels of ROS can operate as signals to promote cell proliferation, whereas high levels of ROS can damage cellular components such as DNA, leading to cell apoptosis (75). Hence, considering that ruthenium(II) complexes and lapachol have been reported as ROS inductor agents, we evaluated the ROS level in cells treated with lapachol-containing ruthenium complexes. Actually, complex (**2**) could also increase general ROS and superoxide anion levels. Interestingly, the cotreatment with ROS inhibitor NAC remarkably suppressed the generation of ROS and prevented the reduction of cell viability. Additionally, complex (**2**) induced DNA damage on DU-145 cells and directly interacts with DNA, suggesting that this compound trigger caspase-dependent apoptosis through ROS-mediated DNA damage in DU-145 prostate cells.

The mechanisms underlying the cytotoxic and antiproliferative activities of complex (**2**) were evaluated at the level of mRNA expression of diverse genes. Notably, it was detected the decreasing expression of genes associated with apoptosis, such as *BCL2* and *BIRC5*, and genes involved in the cell cycle transition, such as *CDK1, CDK4, AURKA, AURKB*, and *AURKC*.

BCL-2 family proteins suppress apoptosis by binding with BAX and BAK proteins that can form pores in the mitochondria and promote the release of cytochrome c, triggering the apoptotic process (76). Lima et al. (77) demonstrated that ruthenium complexes containing phosphine ligands considerably decrease the expression of the *BCL2* gene in sarcoma-180 cells. This effect was associated with the loss of mitochondrial transmembrane potential and activation of caspases. Complex (**2**) also decreased the expression of the *BIRC5* gene on DU-145 cells, which was 6.4 times less expressed than the untreated cells. *BIRC5* encodes a protein called survivin, which became an attractive drug target for anticancer therapies. Studies showed that blocking the transcription of the *BIRC5* gene in prostate cells inhibits the growth of xenografted PC-3 tumors in mice (78). Further, Hurtado et al. (79) reported that copper(II) complexes suppressed the expression of BIRC5 at both mRNA and protein levels in pancreatic cancer cells. Furthermore, complex (**2**) promoted dysregulation of several genes that encode CDKs, especially CDK4, a protein commonly overexpressed in prostate cancer (80). Agents that selectively regulate the activity of the *CDK4* gene have been described in the literature for their ability to inhibit cell proliferation with tolerable toxic effects (81).

We also observed a considerable decrease in the expression of Aurora kinase genes, a family of kinases normally altered in different types of tumors, and consequently, a therapeutic target for cancer (82). Aurora-A and Aurora-B are usually overexpressed in primary prostate cancers and such expression patterns are associate with tumorigenicity, tumor progression, and clinical staging (83). Protein Aurora‐B was also downregulated after treatment with increased concentrations of complex (**2**). Aurora-B plays a crucial role in the Chromosomal Passenger Complex (CPC), a complex necessary for binding of microtubules to the kinetochore of chromosomes, in order to ensure adequate fusion for cell division (84). Addepalli et al. (85) demonstrated that the knockout of the *AURKB* gene mediated by RNAi substantially inhibits the proliferation of PC-3 prostate tumor cells, inducing apoptosis *in vitro*. In fact, other studies have shown that inhibition of Aurora-B has an antiproliferative effect and cause regression of prostate cancer *in vivo* (46, 86, 87). Therefore, complex (**2**) can represent a promising candidate for modulation of *AURKB* expression, opening new trails for anticancer therapy.

It was further observed the upregulation of *MDM4* and *PTGS2* genes in the treated cells. In the present data, complex (**2**) increased the levels of ROS/Superoxide, which could directly modulate the expression of *PTGS2* through the MAPK/ERK cascade on damaged cells (88). Therefore, the increase in *PTGS2* expression is related to the cellular response to high levels of ROS in DU-145 after complex (**2**) exposure. The strength of complex (**2**) generates ROS is also associated with increased expression of *MDM4*, a key regulator of P53 that plays an important role in regulating genomic instability (89). Increased expression of *MDM4* is in agreement with the ROS-mediated DNA damage induced by complex (**2**) as well as the elevated apoptosis by activating the intrinsic (mitochondrial) pathway. Taken together, these results showed that complex (**2**) can regulate several genes associated with the proliferation of prostate adenocarcinoma cells. The regulation of these genes strengthens the mechanism of action of complex (**2**), which appears to be involved in the interruption of the cell cycle in G2/M in response to ROS-mediated DNA damage and the induction of caspase-dependent apoptosis.

In summary, our study revealed that lapachol-containing ruthenium complexes (**1**) and (**2**) exhibited remarkable antiproliferative activities to human prostate adenocarcinoma cells. We found that complex (**2**) significantly induced G2/M cell cycle arrest, induce caspase-dependent apoptosis, and provoke ROS-mediated DNA damage. Moreover, the expression of various genes, including cell division cycle and apoptosis was regulated under treatment. These encouraging results warrant further studies to enhance the knowledge of the anticancer properties of these complexes, as well as identify their pharmacokinetics/pharmacodynamics properties in an attempt to expand the field of prostate anticancer chemotherapy.

## Data Availability Statement

The datasets presented in this study can be found in online repositories. The names of the repository and accession number can be found in the **Supplementary Material**.

## Author Contributions

Conceived and designed the experiments: RG, AB and FP. Performed the synthesis and structural characterization of the novel complexes: KO and AG. Performed the cellular and molecular effects of the novel complexes: RG, PS, and AA. Analyzed the data and wrote the manuscript: RG, PS, AA, and KO. Provided the technical and financial support: RG, AB, and FP. All authors contributed to the article and approved the submitted version.

## Conflict of Interest

The authors declare that the research was conducted in the absence of any commercial or financial relationships that could be construed as a potential conflict of interest.
