# Supplementary material for: A Novel Ruthenium(II) Complex With Lapachol Induces G2/M Phase Arrest Through Aurora-B Kinase Down-Regulation and ROS-Mediated Apoptosis in Human Prostate Adenocarcinoma Cells
Source: Front Oncol. 2021 Jun 24;11:682968. doi: 10.3389/fonc.2021.682968 (PMC8264259; doi:10.3389/fonc.2021.682968)
Supplement: Supplementary file 1 [file DataSheet_1.docx]

Supplementary Material

|  |  | ***Cancer cells*** | | | | | | |
| --- | --- | --- | --- | --- | --- | --- | --- | --- |
| ***Non-cancer cells*** |  | **A-375** | **A549** | **Caco-2** | **DU-145** | **HepG2** | **MDA-MB-231** | **PC-3** |
| **FGH** | **(1)** | 1.2 | 0.9 | 1.5 | 3.4 | 1.8 | 1.5 | 2.4 |
|  | **(2)** | 2.7 | 1.0 | 2.0 | 3.1 | 1.7 | 1.2 | 2.2 |
|  | **CIS** | 0.7 | 0.9 | 0.6 | 0.5 | 0.8 | 0.5 | 1.0 |
|  | **DXR** | 0.7 | 1.4 | 1.2 | 1.1 | 1.1 | 1.2 | 1.0 |
| **PNT-2** | **(1)** | 2.4 | 1.8 | 2.9 | 6.5 | 3.5 | 2.8 | 4.5 |
|  | **(2)** | 7.5 | 2.6 | 5.7 | 8.5 | 4.5 | 3.4 | 6.2 |
|  | **CIS** | 0.6 | 0.8 | 0.5 | 0.4 | 0.7 | 0.4 | 0.9 |
|  | **DXR** | 0.9 | 1.7 | 1.5 | 1.4 | 1.3 | 1.5 | 1.2 |

**Supplementary Table 1. Selectivity index of the Ru complexes**

Data are presented with the selectivity index (SI) calculated using the following formula: SI = IC_50_[non-cancer cells]/IC_50_[cancer cells]. Cancer cells: A-375 (human malignant melanoma); A549 (human lung carcinoma); Caco-2 (human colorectal adenocarcinoma); DU-145 (human prostate adenocarcinoma); HepG2 (human hepatocellular carcinoma); MDA-MB-231 (human breast adenocarcinoma) and PC-3 (human prostate adenocarcinoma). Non-cancer cells: FGH (human mouth fibroblast) and PNT-2 (human prostate epithelial cells). Cisplatin (CIS – Fauldcispla^®^) and doxorubicin (DXR – Fauldoxo^®^) were used as positive controls.

**Supplementary Figure 1.** Infrared spectrum of the complex (**1**), [Ru(Lap)(dppm)(bipy)]PF_6_, in KBr pellets.

**Supplementary Figure 2.** Infrared spectrum of the complex (**2**), [Ru(Lap)(dppm)(phen)]PF_6_, in KBr pellets.


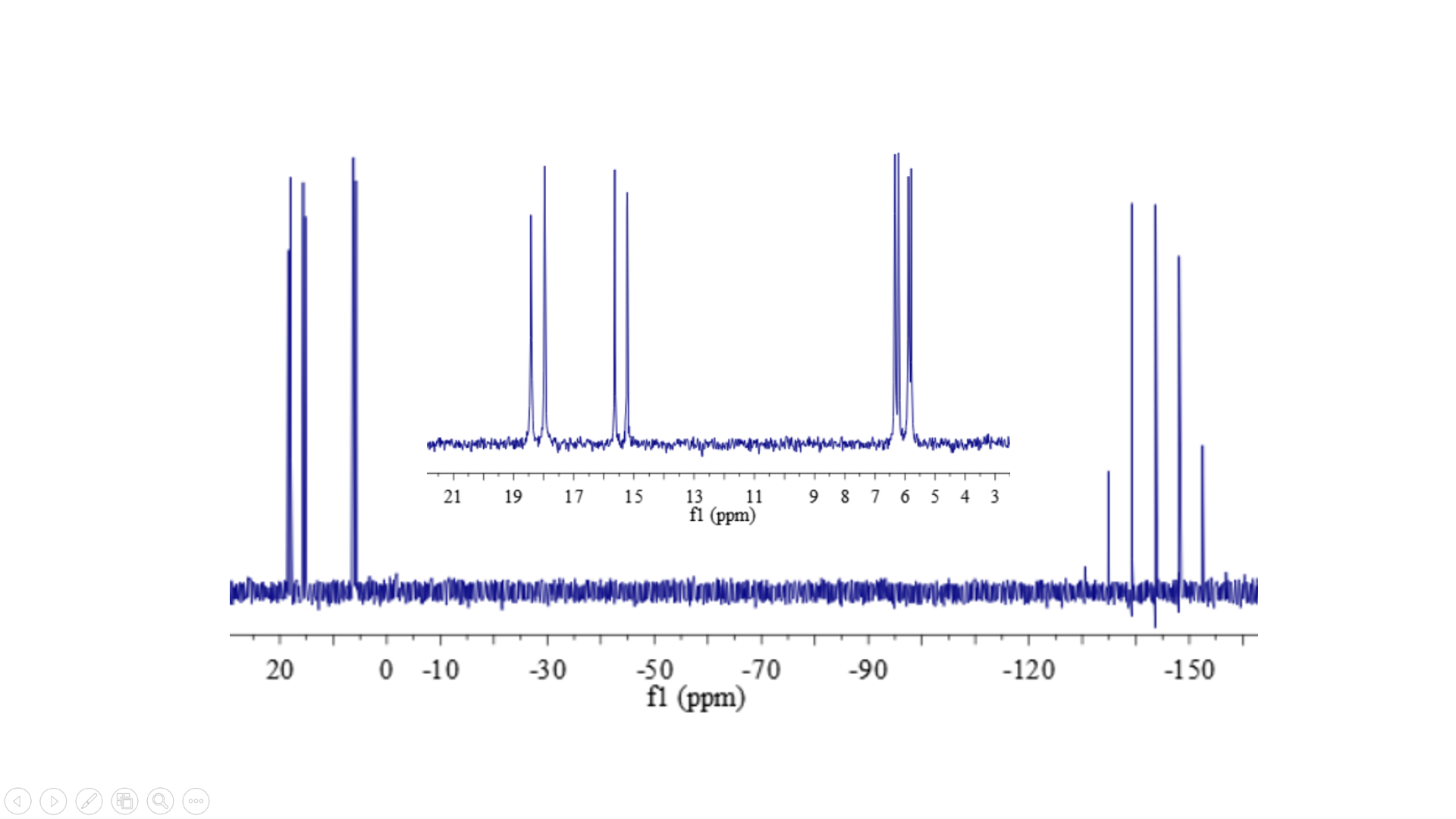


**Supplementary Figure 3**. ^31^P{^1^H} NMR of complex (**1**) in DMSO at 25 ºC.


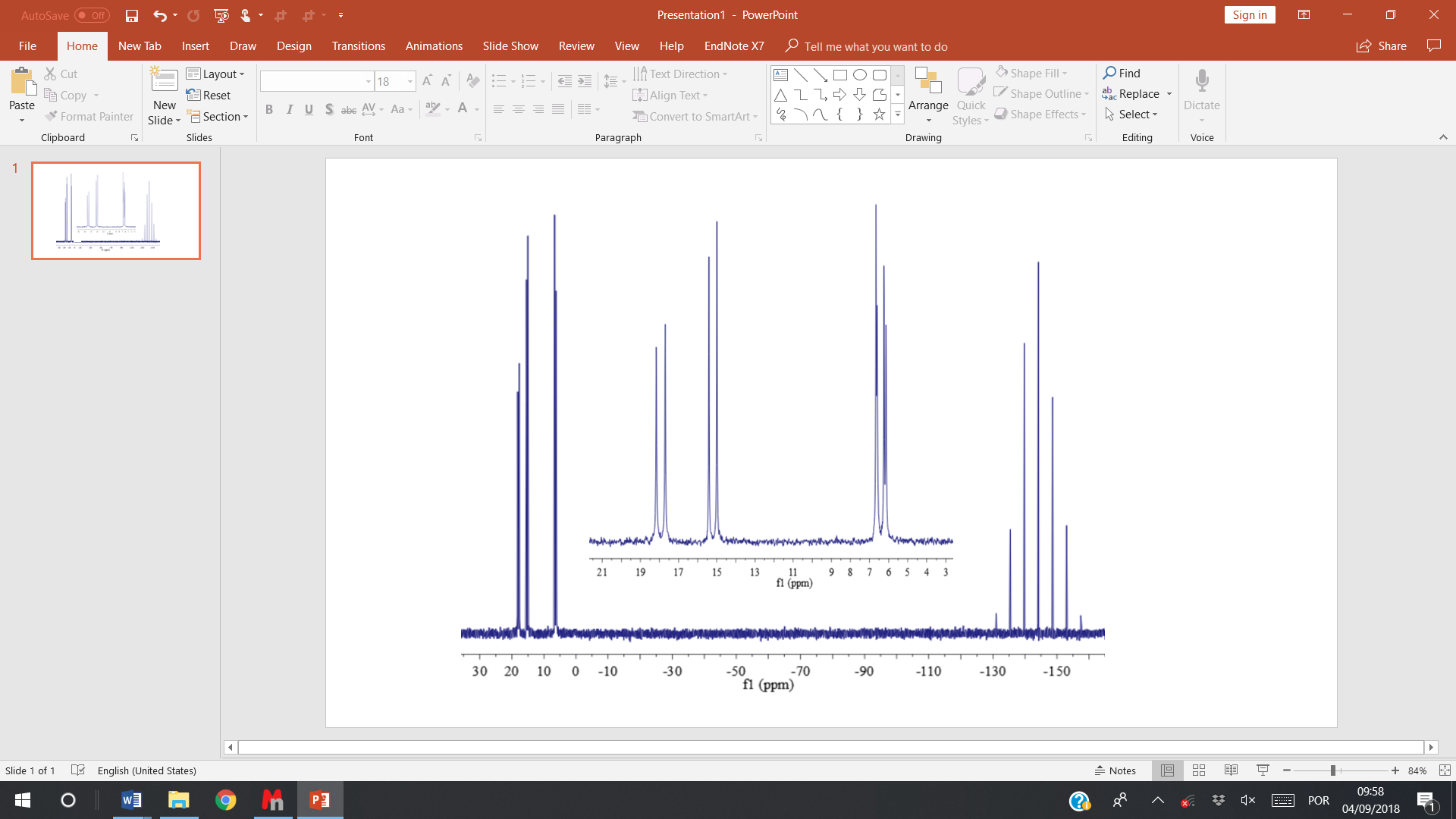


**Supplementary Figure 4**. ^31^P{^1^H} NMR of complex (**2**) in DMSO at 25 ºC.

**
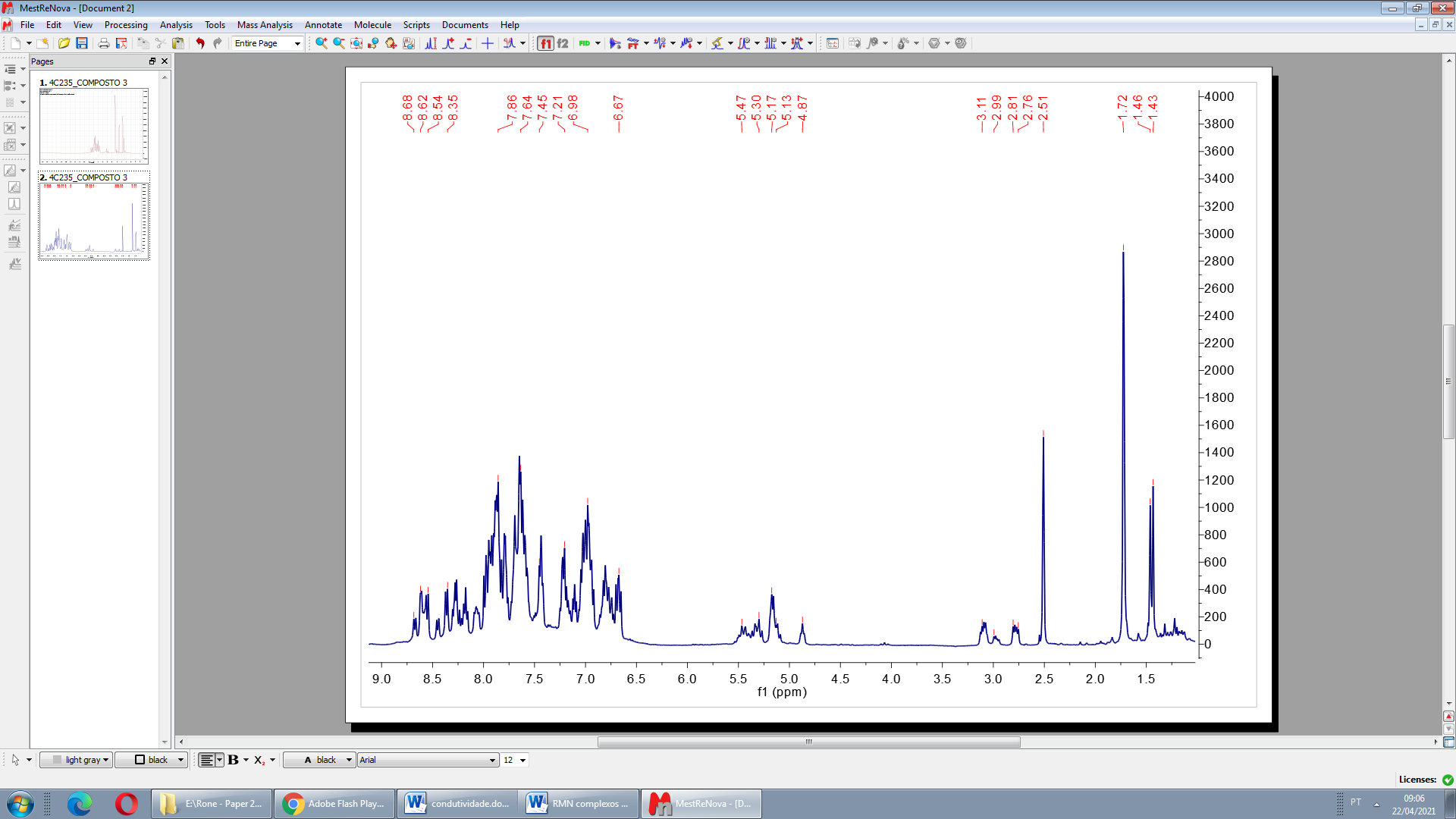
**

**Supplementary Figure 5.** ^1^H NMR spectrum of complex (**1**) in DMSO-d_6_.


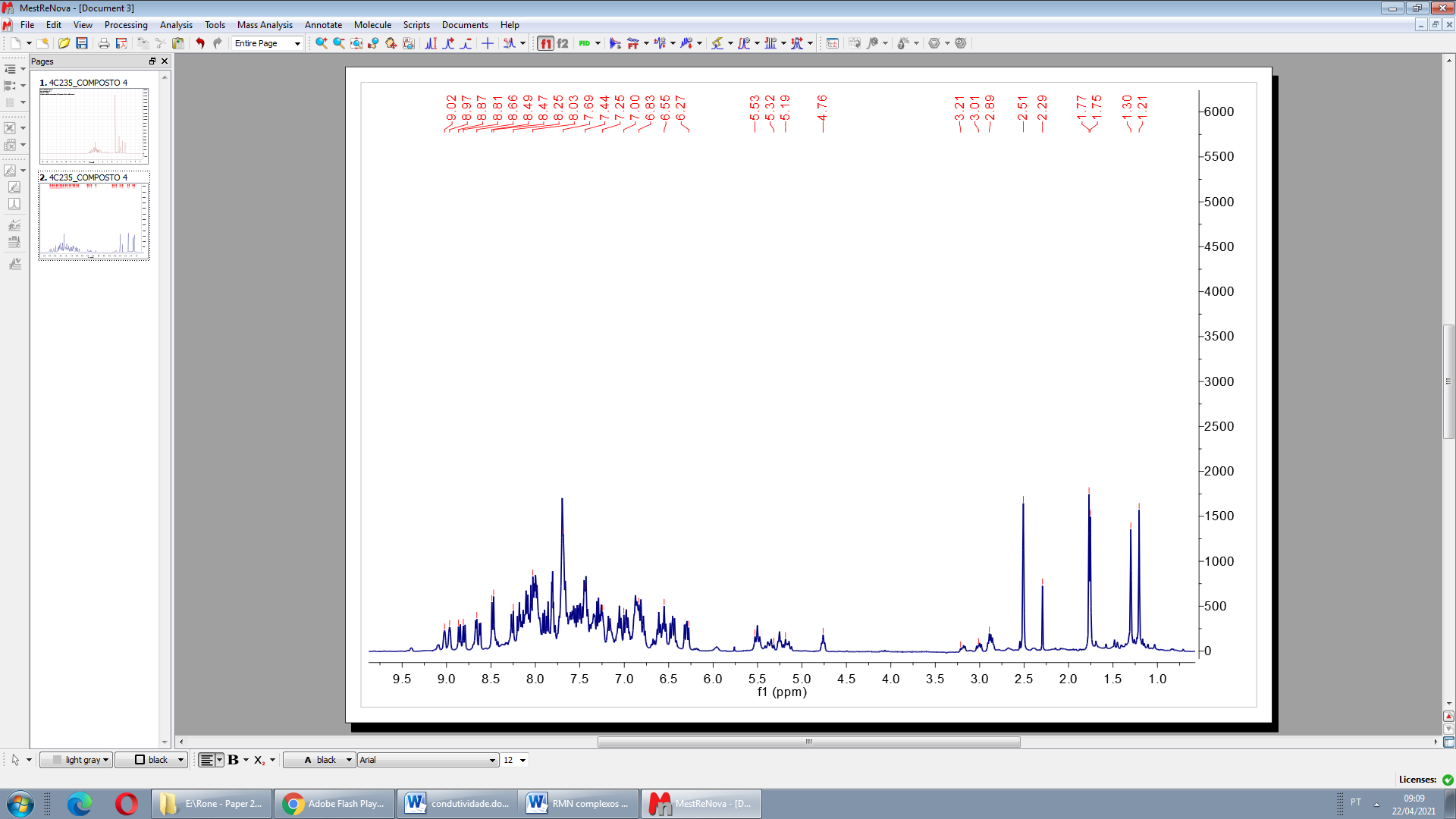


**Supplementary Figure 6.** ^1^H NMR spectrum of complex (**2**) in DMSO-d_6_.

**
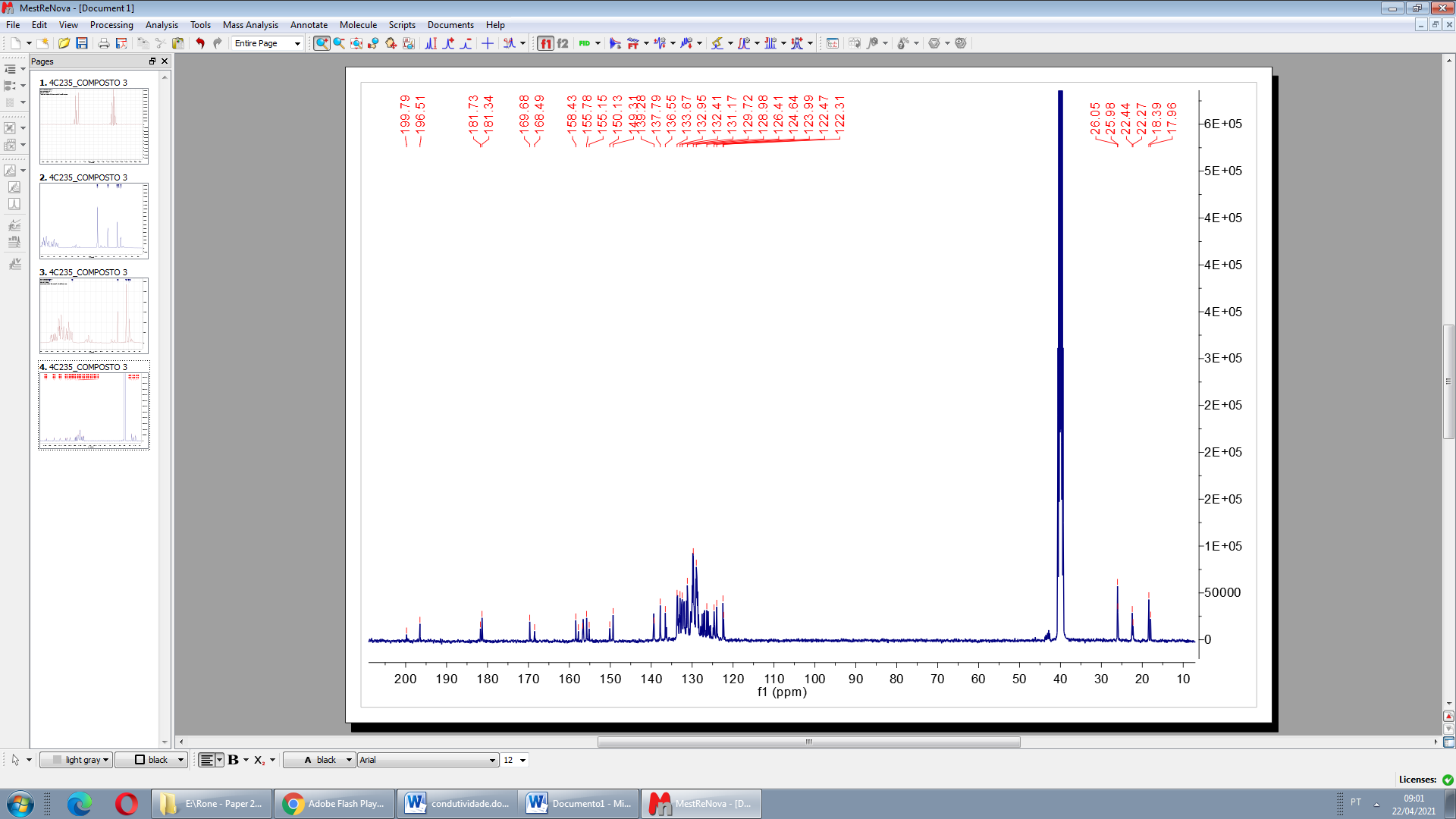
**

**Supplementary Figure 7.** ^13^C{^1^H} NMR spectrum of complex (**1**) in DMSO-d_6_.


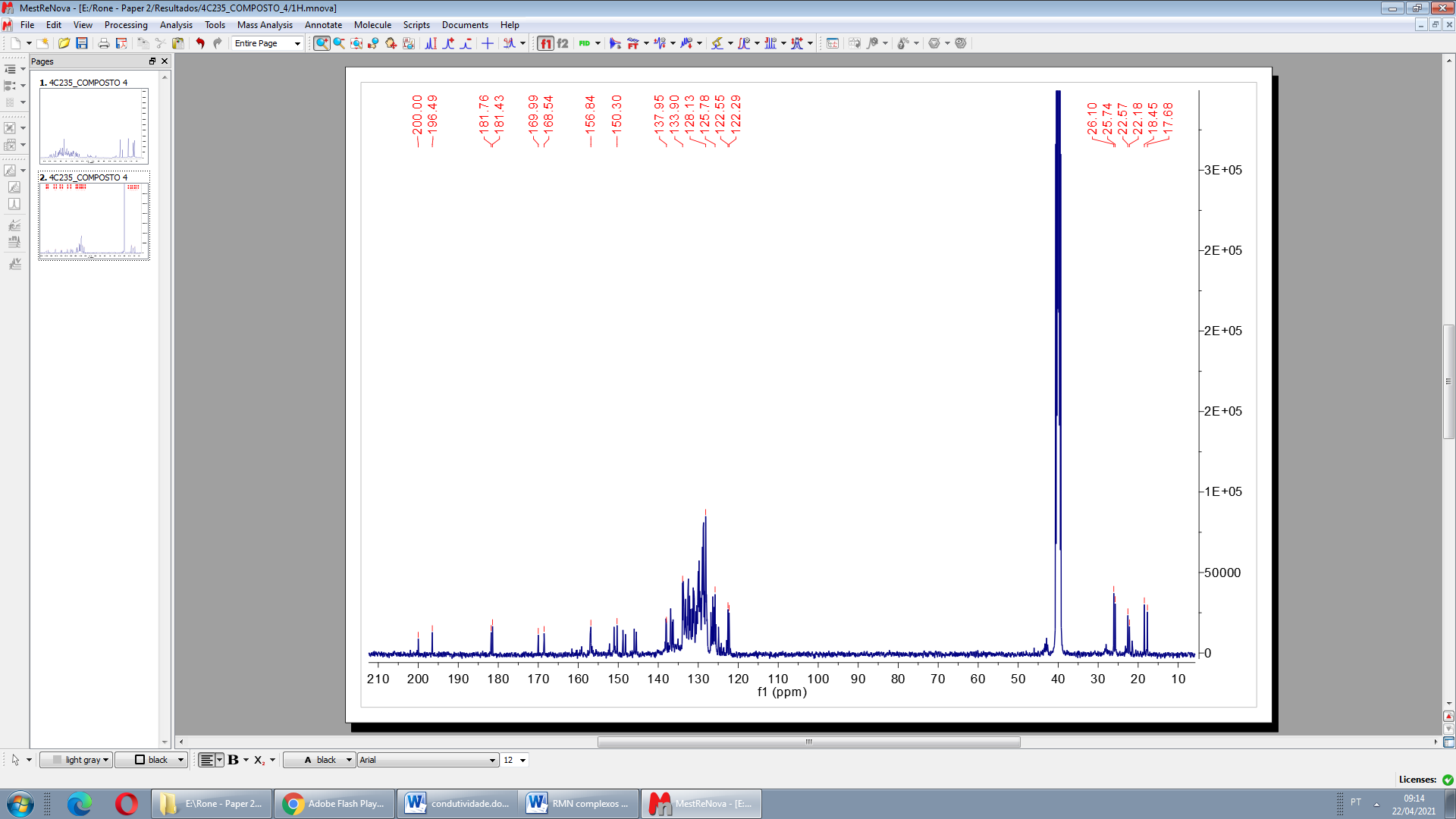


**Supplementary Figure 8.** ^13^C{^1^H} NMR spectrum of complex (**2**) in DMSO-d_6_.


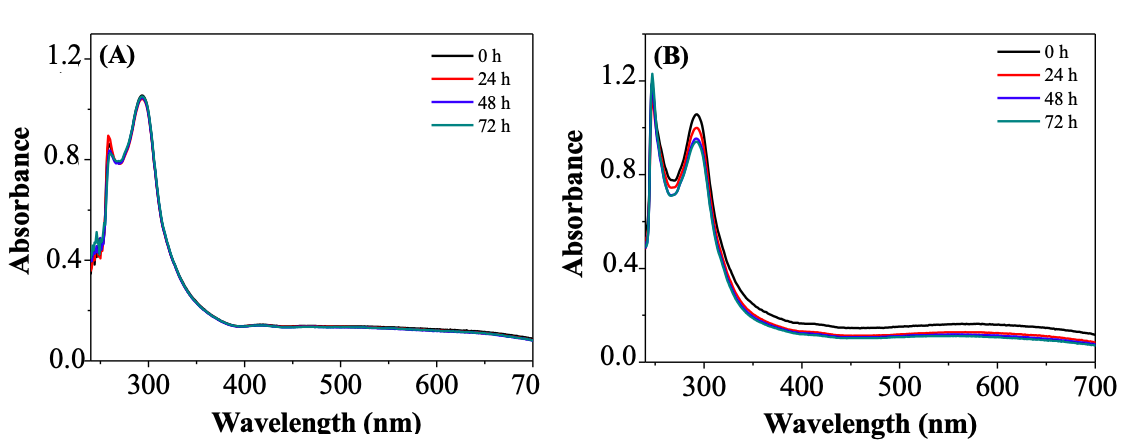


**Supplementary Figure 9**. UV-Vis spectra of complex (**1**) in (**A**) DMSO and (**B**) DMSO/Tris-HCl buffer (pH 7.4) (50:50, v/v), at different times.


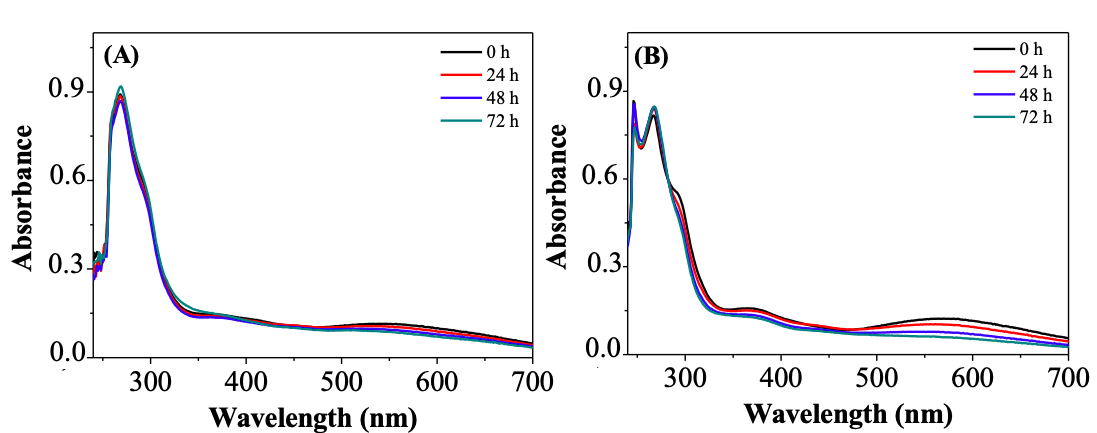


**Supplementary Figure 10**. UV-Vis spectra of complex (**2**) in (**A**) DMSO and (**B**) DMSO/Tris-HCl buffer (pH 7.4) (50:50, v/v), at different times.

**
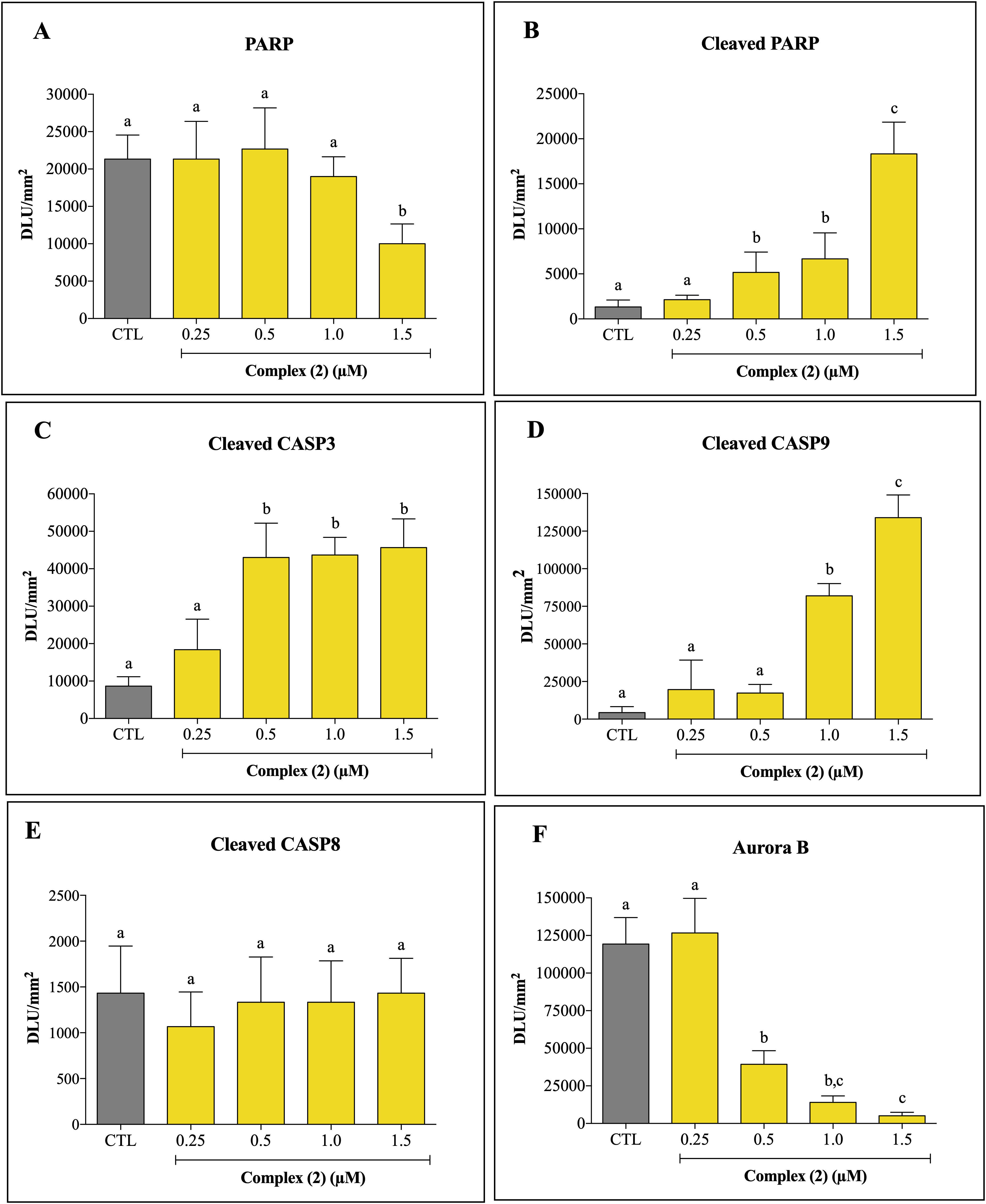
**

**Supplementary Figure 11.** Densitometric analysis of band gray optical density of proteins PARP (A), cleaved PARP (B), cleaved CASP3 (C), cleaved CAS9 (D), cleaved CASP8 (E) and Aurora B (F), expressed as digital light units per square millimeter (DLU/mm^2^). The negative control (CTL) was treated with the vehicle (DMSO 0.1% v/v) used for diluting the complex. Data are presented as the mean ± S.E.M. of three independent experiments. Values not sharing the same letter are significantly different from each other (*p* < 0.05; ANOVA followed by the Tukey’s test).

**
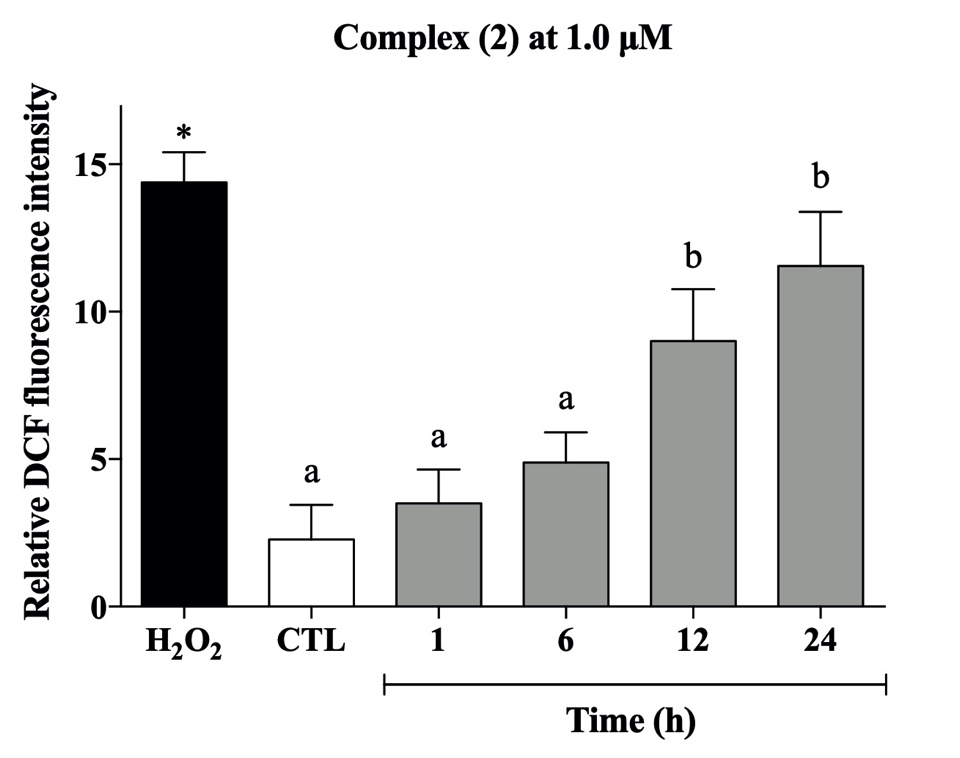
**

**Supplementary Figure 12.** ROS levels of DU-145 cells after 1, 6, 12 and 24 h of incubation with complex (**2**) at IC_50_ (1.0 μM). The negative control (CTL) was treated with the vehicle (0.1% DMSO) used for diluting the tested compound. Hydrogen peroxide (H_2_O_2_, 100 μM) was used as the positive control. Data are presented as the mean ± S.E.M. of three independent experiments performed in triplicate or quadruplicate. **p* < 0.05 compared with the control by ANOVA followed Dunnet’s test. Values not sharing the same letter are significantly different from each other (*p* < 0.05; ANOVA followed by the Tukey’s test).

**
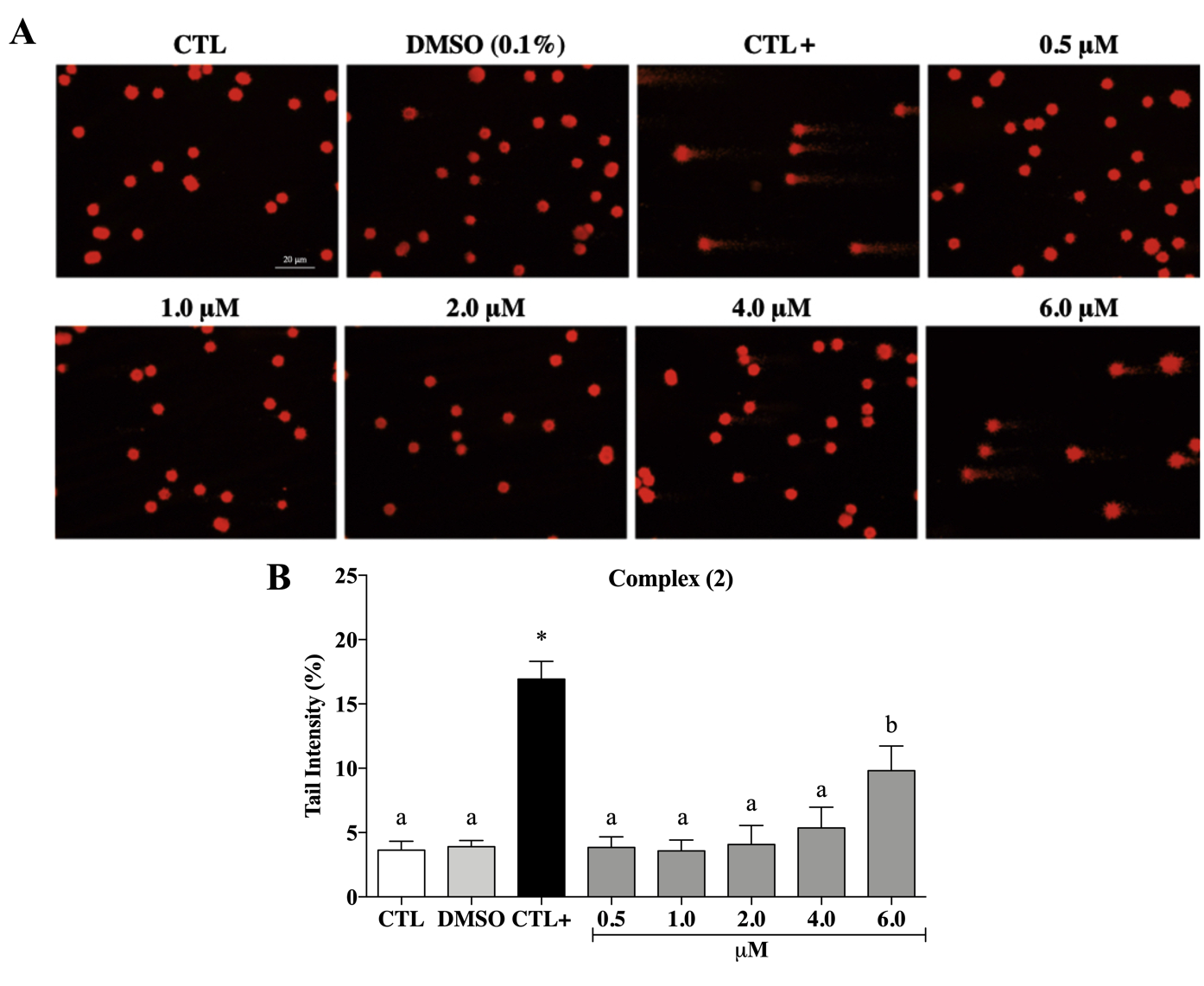
**

**Supplementary Figure 13.** **Genotoxic effect modulated by ruthenium complex (2) in PNT-2 cells.** (A) Representative image samples obtained by application of alkaline version of comet assay on PNT-2 cells treated with complex (**2**) at 0.5, 1.0, 2.0, 4.0 and 6.0 μM for 24 h. (B) Bar graph represents the tail intensity (%), which is directly correlated to DNA damage. The images were acquired with fluorescence microscopy at a total magnification of 400x (scale bar = 20 μm) and correspond to a representative assay from three independent experiments. The negative control (CTL) represents the untreated cells, DMSO (0.1% (v/v)) was the vehicle used for diluting the tested compound and the positive control (CTL+) was treated with methyl methanesulfonate (150 μM). Data are presented as the mean ± S.E.M. of three independent experiments. **p* < 0.05 compared with the control by ANOVA followed Dunnet’s test. Values not sharing the same letter are significantly different from each other (*p* < 0.05; ANOVA followed by the Tukey’s test).


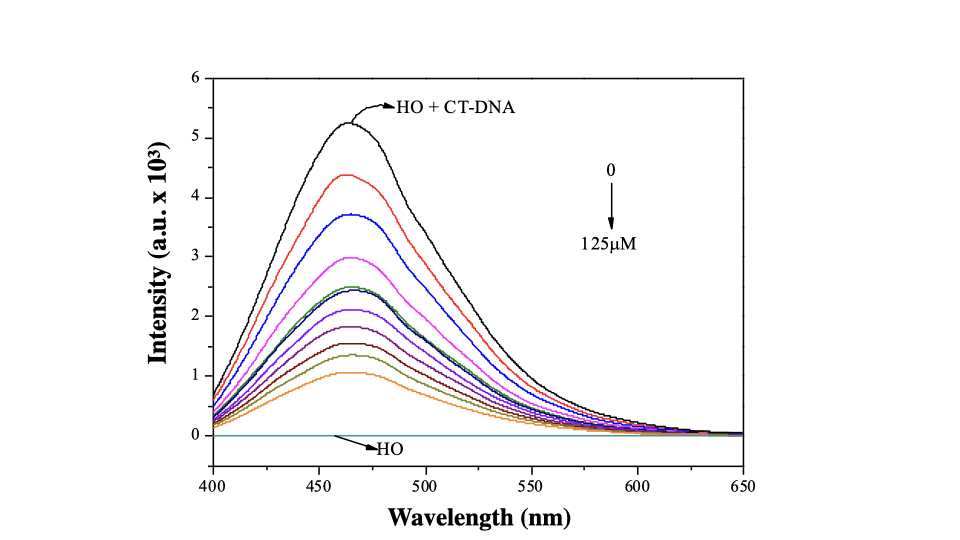


**Supplementary Figure 14.** Emission spectra of Hoechst (2.7 µM, λ_ex_ = 343 nm), CT-DNA (125 µM) in presence of complex (**1**) in different concentrations (0 - 125 µM), at 37 °C.

**The datasets presented in this study can be found online in the UNESP Institutional Repository Available at: <http://hdl.handle.net/11449/191678>.**
